# Preparation and Characterization of Functional Composite Polymeric Media Based on Potassium Polyacrylate Hydrogel for Root-Zone Applications

**DOI:** 10.3390/polym18202592

**Published:** 2026-10-21

**Authors:** Dmitry Khort, Alexey Kutyrev, Igor Smirnov, Nikolay Maistrenko, Rostislav Filippov, Andrey Chilikin

**Affiliations:** 1 Federal Scientific Agroengineering Center VIM, 109428 Moscow, Russia; dmitriyhort@mail.ru (D.K.); rashn-smirnov@yandex.ru (I.S.); rostislav-filippov@yandex.ru (R.F.); gwinnyandrei@yandex.ru (A.C.); 2 Russian State Agrarian University—Moscow Timiryazev Agricultural Academy, 49, Timiryazev Street, 127550 Moscow, Russia; nmaystrenko@rgau-msha.ru

**Keywords:** potassium polyacrylate hydrogel, composite polymeric media, vermicompost, perlite, fraction-dependent properties, ion exchange, sustained potassium release, water retention, Raman spectroscopy, root-zone application

## Abstract

Functional composite polymeric media based on superabsorbent hydrogels are promising materials for controlled water retention and ion-exchange nutrient release in root-zone applications. This study investigates the design, fraction-dependent properties, and functional performance of composite polymeric media based on potassium polyacrylate hydrogel (B 415 K) combined with vermicompost and perlite. Hydrogel fractions (fine, medium 0.5–1.5 mm, and coarse) were characterized by optical microscopy and Raman spectroscopy (785 nm). Raman analysis revealed six characteristic bands (1086, 710, 673, 531, 282, and 157 cm^−1^) for the medium fraction, and swelling ratios followed the order: fine (23.4 g/g) > medium (19.1 g/g) > coarse (12.7 g/g). The medium fraction provided the optimal balance between hydration, structural integrity, and sorption of organic growth stimulants, establishing structure–property–performance relationships for the composite polymeric media. Composite media were prepared at a dry-component ratio of hydrogel:vermicompost:perlite = 1:40:20, and their water retention, pH buffering, electrical conductivity, and K^+^ release dynamics were evaluated over 21 days. The composite medium maintained stable moisture at 68–72% of full water-holding capacity, whereas the control decreased to 55–60%. Exchangeable K^+^ remained at 120–135 mg/L throughout the experiment, whereas in the control it decreased from 100 to 65 mg/L, confirming the stimuli-responsive ion-exchange function of the polyacrylate network. The performance of the polymeric media was demonstrated using garden strawberry (*Fragaria* × *ananassa* ‘Alba’) rosettes as a model root-zone application: rooting reached 100% in the composite medium vs. 86% in the control and 92% in pure hydrogel; total root system length increased 1.6-fold, and root dry biomass increased by 45%. Field validation confirmed the stability of the polymeric medium under open-ground conditions, with 96.7% survival and a 30% reduction in irrigation frequency. The developed composite polymeric media provide sustained water retention and potassium release without additional mineral fertilizers, demonstrating the potential of rational design of functional polymeric materials for root-zone application.

## 1. Introduction

Functional polymeric materials capable of retaining water and releasing nutrients in a controlled manner are of increasing interest for root-zone applications [1,2]. Among them, superabsorbent hydrogels based on cross-linked polyacrylates are widely studied because of their high water-absorbing capacity, tunable swelling behavior, and ability to act as reservoirs for ions and bioactive compounds [3,4,5]. However, the design of composite polymeric media that combine a synthetic hydrogel with organic and mineral fillers, and the relationship between their fraction-dependent structure and functional performance, remain insufficiently understood. This study addresses this gap by developing and characterizing composite polymeric media based on potassium polyacrylate hydrogel, vermicompost, and perlite, and by demonstrating their performance in a model root-zone application.

The transition to sustainable agriculture requires the development of technologies that minimize the use of water and mineral resources while maintaining crop productivity. Strawberry (*Fragaria* × *ananassa*), as a high-value berry crop, has high water requirements, which, under conditions of climate change and freshwater scarcity, makes the development of artificial substrates with a regulated water regime particularly relevant.

Garden strawberry (*Fragaria* × *ananassa*) is one of the world’s most economically significant and intensively cultivated berry crops, contributing substantially to agricultural revenues and being valued for its nutritional and functional properties, including their content of vitamin C, phenolic compounds, anthocyanins, tannins, and dietary fiber. Modern cultivation systems are characterized by high resource intensity: the crop has high requirements for water supply, mineral nutrition, protection against diseases and pests, as well as protected cultivation infrastructure. Taken together, these factors result in a significant environmental footprint due to greenhouse gas emissions, the use of non-renewable resources, and local impacts on ecosystems [6,7,8]. These high resource demands make the optimization of substrate cultivation a critical factor for sustainable strawberry production.

A key element of sustainable substrate cultivation is the optimization of mineral nutrition and nutrient solution composition with respect to plant developmental stages [9,10]. Widely used standard formulations, such as the Yamazaki solution, are often applied without modification, which does not account for changes in plant requirements over time [11]. Studies on commercial substrates have shown that adjusting the N:K and K:Ca ratios, as well as the proportions of nitrate and sulfate ions, can improve nutrient use efficiency [12,13,14]. The modified formulations increased photosynthetic activity and berry yield by 20–26%, improved organoleptic and biochemical fruit quality, and provided more stable pH buffering, indicating that strawberries have increased nitrogen and calcium requirements and a rising relative potassium demand during flowering and fruit filling [15,16,17].

Traditional open-field cultivation on well-drained loam soils (pH 5.5–6.5) with mulch and split nitrogen application is subject to yield fluctuations due to climate variability, pesticide use, and soil-borne diseases [14,18,19]. Protected cultivation expands the geographical range and reduces pesticide use but increases material and energy costs [18,20,21].

Beyond these economic and technological constraints, water supply remains a critical challenge in hot climates. Hydroponic and substrate-based cultivation in hot climates faces challenges related to water supply and heat stress. In tropical and subtropical regions, high daytime temperatures and atmospheric moisture deficit impair strawberry growth and gas exchange, reducing photosynthesis and productivity [22,23]. In this regard, the incorporation of water-retaining polymers (hydrogels) into the substrate, which are capable of accumulating and slowly releasing moisture into the root zone, is considered a promising approach [24].

Physiologically, hydrogels can also affect gas exchange: at later vegetation stages, increasing hydrogel doses were reported to reduce stomatal conductance and transpiration, presumably because part of the water becomes bound within the polymer matrix [25,26].

From an agronomic perspective, incorporating hydrogel into the substrate creates a more buffered moisture supply regime, reduces the amplitude of moisture fluctuations, and decreases the dependence of plant functions on irrigation frequency. This allows optimization of fertigation and irrigation regimes and reduces the risk of acute water stress, which is particularly important for regions with limited water resources [9,27]. To address these requirements, functional polymeric materials with tunable water-retention and ion-exchange properties are required.

From a polymer science perspective, potassium polyacrylate hydrogels belong to the class of cross-linked polyelectrolytes whose swelling behavior is governed by the balance between osmotic pressure of counterions, polymer–solvent interactions, and elastic retraction of the network. Their functional performance depends on cross-linking density, charge density, particle size, and the presence of fillers. When such hydrogels are combined with organic and mineral components, the resulting composite polymeric media exhibit coupled water retention, ion-exchange, and aeration functions. The ion-exchange capacity of the polyacrylate network, originating from carboxylate groups, allows the material to act as a reservoir of K^+^ ions and to buffer the ionic composition of the surrounding solution. Understanding the relationship between the fraction size, swelling behavior, and sorption of bioactive compounds is therefore essential for the rational design of composite polymeric media for root-zone applications. Crucially, these materials are not passive water reservoirs. The polyacrylate network can be engineered to function as a stimuli-responsive carrier, where the release of ions (such as K^+^) is governed by the ionic strength and pH of the surrounding root-zone solution. This transforms the hydrogel from a simple water-retention agent into a functional polymeric medium with coupled water-retention and ion-exchange properties. Furthermore, the incorporation of organic (vermicompost) and mineral (perlite) fillers is not merely a physical mixture but a design strategy to create a composite polymeric medium. The filler particles can act as physical cross-linking points, influence the swelling behavior of the network, and introduce additional functionalities such as aeration and microbial support, which are not achievable with the hydrogel alone.

Depending on the origin of the polymer, agricultural hydrogels are classified as synthetic and biodegradable/hybrid. The most widely used materials are copolymers of acrylic acid and acrylamide (sodium and potassium polyacrylates), sometimes with the addition of acrylonitrile, vinyl sulfonates, and other components [28]. Cellulose hydrogels are produced by chemical cross-linking (e.g., with epichlorohydrin, di- or triphosphates) or by physical cross-linking of soluble derivatives such as carboxymethyl cellulose. Alginate hydrogels are formed through ionic cross-linking of Ca^2+^ ions with blocks of hyaluronic acid (calcium alginate). The most promising materials for sustainable agriculture are semi-synthetic hydrogels in which natural polymers (starch, cellulose, chitosan, alginate, lignin) are modified with grafted chains of synthetic monomers [29,30].

The most relevant applications of hydrogel-based media include the following:

1. The pre-sowing or root-zone application of hydrogel in the form of granules or powder, as well as seed treatment, makes it possible to create a stable reservoir of moisture and nutrients directly within the root development zone, providing critical support during the early stages of ontogenesis [31,32].
2. The incorporation of fillers with specified properties (sorbents, microorganism carriers, and aerating additives) into the hydrogel matrix [33,34] makes it possible to create multicomponent systems with a combined effect, such as simultaneous moisture retention, nutrient delivery, and biological control of pathogens.
3. The selection of the polymer type, cross-linking density, composition, and component ratio makes it possible to precisely control the rate and triggers (pH, moisture, and enzymatic activity) of active substance release, adapting the system to a specific crop and soil type [35].

However, the potential of composite media combining a synthetic hydrogel, organic vermicompost, and a mineral loosening component (perlite) for the rooting stage of strawberry rosettes remains insufficiently studied. Most studies use pure vermicompost, which is known to have excessive density, insufficient aeration, and an excessive concentration of nutrients, which may inhibit root formation. Data on the effect of the fractional composition of potassium polyacrylate hydrogel on the immobilization of organic growth stimulants, as well as on the prolonged release of potassium from the polymer matrix itself without the application of mineral fertilizers, are lacking. The present study addresses this gap by proposing a scientifically substantiated composite medium consisting of hydrogel (medium fraction, 0.5–1.5 mm), vermicompost, and perlite at a dry-component ratio of 1:40:20, with a detailed assessment of the dynamics of moisture, pH, exchangeable potassium, and root system morphometric parameters, including third- and fourth-order roots, during the initial stage of vegetation of *Fragaria* × *ananassa*. Thus, the targeted design and application of hydrogel-based artificial media represent a promising scientific and technological approach to the transition toward smart and sustainable agriculture, simultaneously addressing the objectives of increasing productivity, conserving resources, and minimizing anthropogenic impacts on the environment.

The scientific novelty of this work is threefold. First, the effect of the potassium polyacrylate hydrogel fraction (fine, medium, and coarse) on the sorption of organic growth stimulants was systematically studied by Raman spectroscopy, and the medium fraction (0.5–1.5 mm) was identified as providing the optimal balance between hydration, structural integrity, and sorption capacity. Second, a new composite polymeric medium based on potassium polyacrylate hydrogel, vermicompost, and perlite (1:40:20 by dry weight) was developed, which combines water-retention and ion-exchange functions. Third, the dual functional behavior of the polyacrylate network—simultaneous water retention and sustained K^+^ release through the ion-exchange mechanism—was demonstrated over 21 days without additional mineral fertilization, confirming the structure–property–performance relationships for the developed composite polymeric media.

It should be emphasized that the term “composite polymeric media” used in this work refers to composite hydrogel-based polymeric materials designed for root-zone application, in which the potassium polyacrylate hydrogel acts as the functional polymeric matrix, vermicompost as the organic filler, and perlite as the mineral aeration component. This definition is used consistently throughout the manuscript.

Section 2 describes the process of developing the composite polymeric media. Spectral and elemental analyses based on Raman microscopy (785 nm) were used to assess the immobilization of organic compounds on different hydrogel fractions (fine, medium, and coarse). Section 3 presents the dynamics of the agro-physical parameters, including substrate moisture, pH, and K^+^ concentration. Root system morphometry was performed, and the physiological status of the plants was analyzed based on leaf chlorophyll content. Section 4 confirms the advantages of the composite medium due to its stable moisture regime, characterized by a narrow moisture range that eliminates water stress and increases rooting to 100%, and explains the effect of prolonged K^+^ release from the hydrogel polymer matrix acting as an ion-exchange reservoir that stabilizes plant nutrition. The relationship with the United Nations Sustainable Development Goals (SDGs 6, 12, and 15) is associated with reduced water consumption, decreased fertilizer leaching, and improved resource efficiency.

## 2. Materials and Methods

### 2.1. Preparation and Characterization of Composite Polymeric Media

#### 2.1.1. Hydrogel Fractionation and Characterization

Potassium polyacrylate hydrogel (B 415 K) was fractionated into fine, medium (0.5–1.5 mm), and coarse fractions to study the effect of particle size on swelling behavior, structural integrity, and sorption of organic compounds. Hydration was performed in distilled water at 5 g/L for 5 h, after which the hydrated fractions were placed in a plastic tube (3.5 cm diameter, 10 cm length) with a fine-mesh nylon net at the bottom. The tube was immersed in a solution of a combined organic growth stimulant (thiamine, succinic acid, and heteroauxin) for 5 min to evaluate the sorption capacity of each fraction. The tube was then transferred to distilled water, excess liquid was removed, and the sample was left for 20 min. Samples were collected at distances of 2, 4, and 6 cm from the bottom of the tube in triplicate, and the resulting values were averaged. The swelling ratio was determined in distilled water in order to characterize the intrinsic swelling capacity of the polyacrylate network; it should be noted, however, that in the rhizosphere the swelling behavior is additionally governed by the ionic strength and composition of the soil solution, and that the presence of hardness ions (Ca^2+^, Mg^2+^) above 2 mmol/L reduces the water absorption of potassium hydrogels, as noted in Section 2.1.2.

This hydration step is considered a stage of polymeric matrix preparation, and the subsequent sorption of organic growth stimulants is interpreted as immobilization of bioactive compounds in the polymer network.

**Figure 1:**
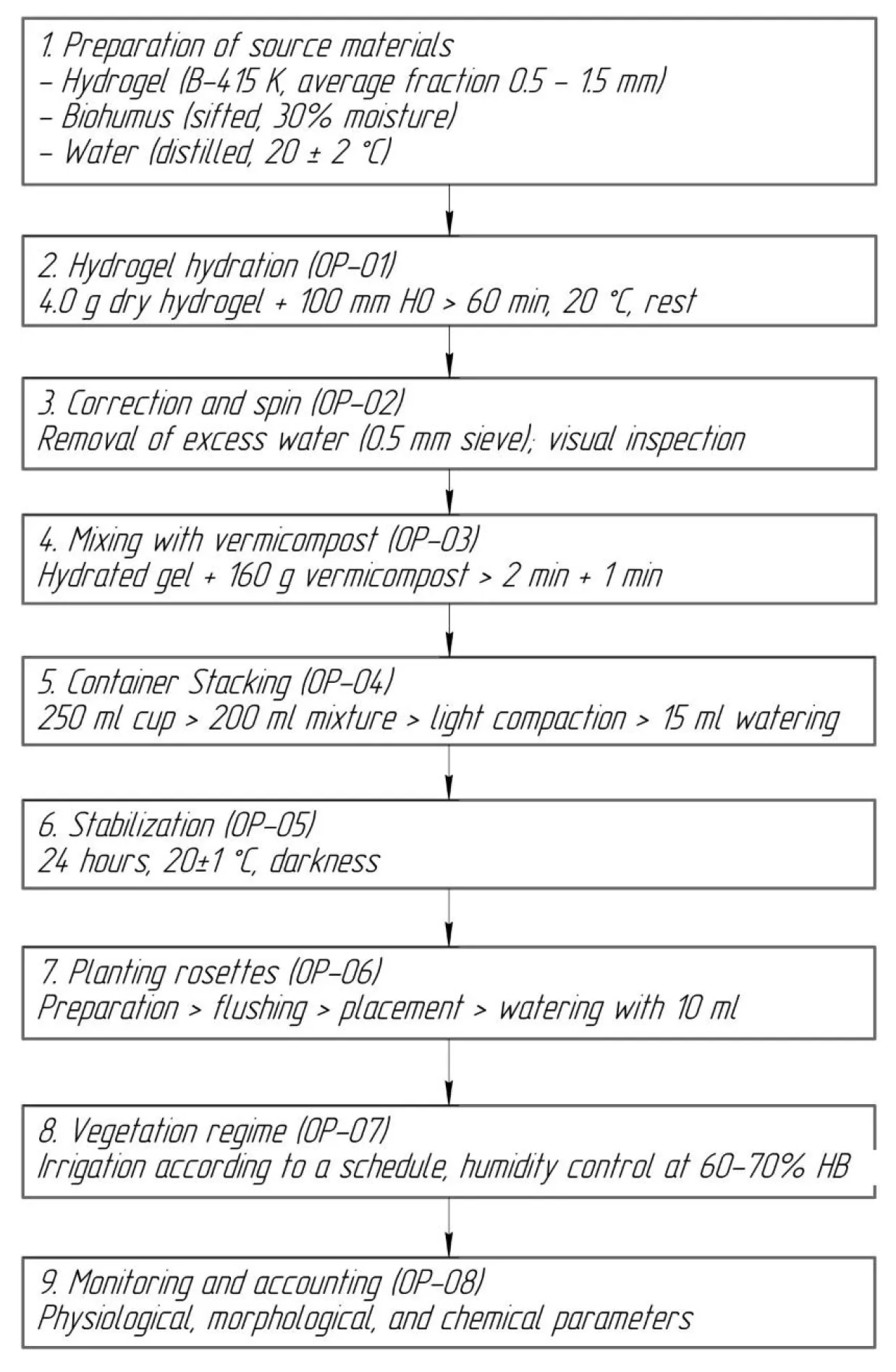
Preparation of the composite polymeric medium.

The process of creating the composite polymeric medium consisted of eight technological operations and took 25–32 h (Figure 1).

**Figure 2:**
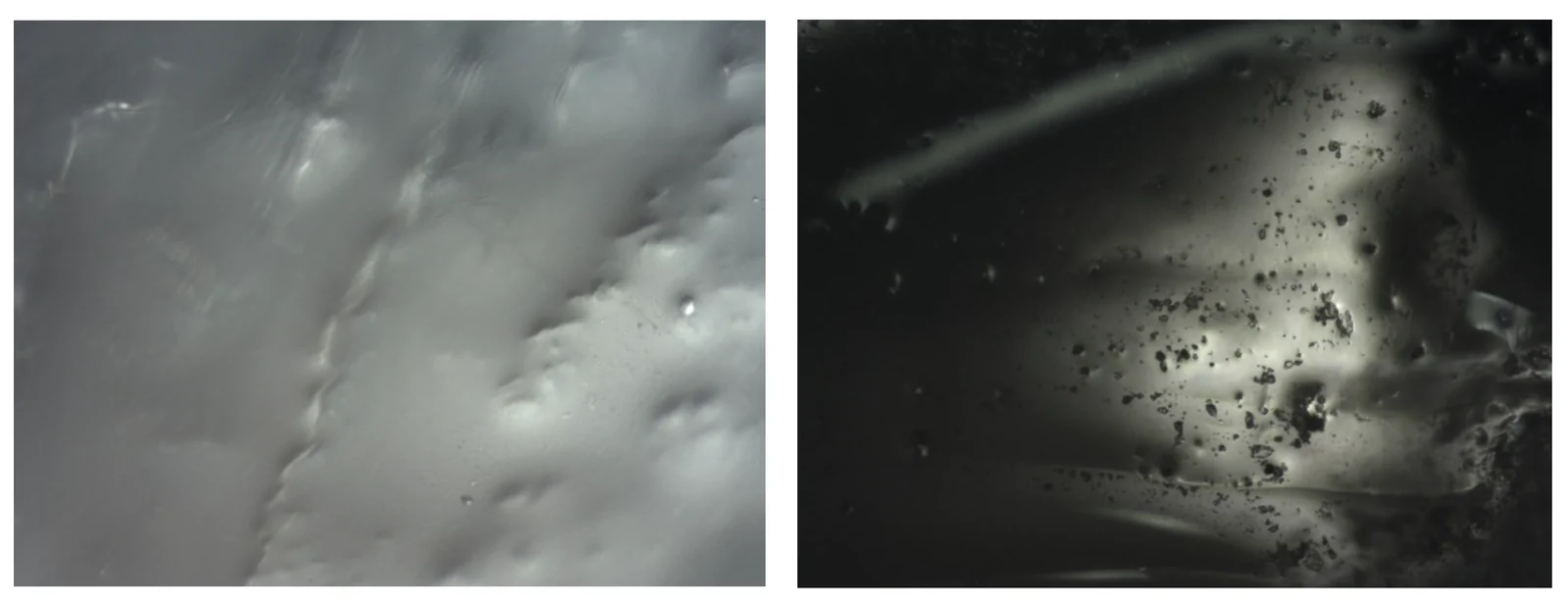
Fine-grained fraction. Green spectrum.

**Figure 3:**
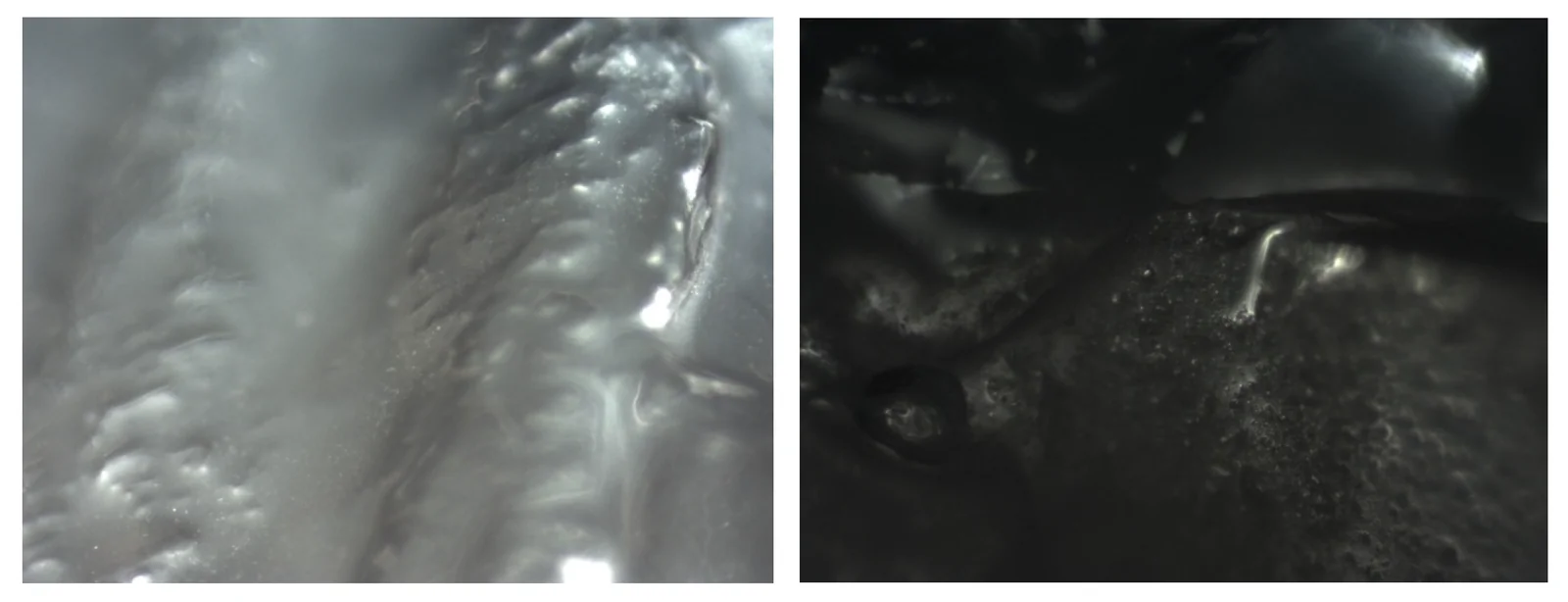
Medium fraction. Blue spectrum.

**Figure 4:**
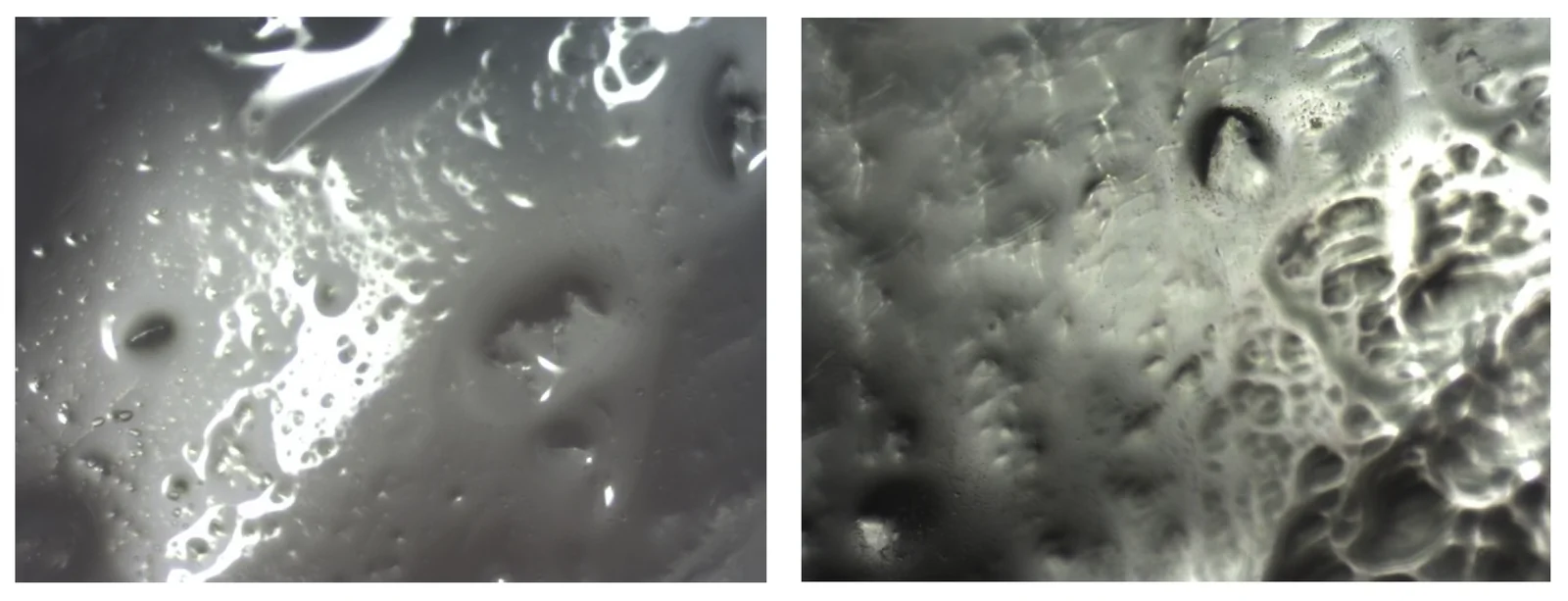
Coarse fraction. Red spectrum.

Based on visual assessment using an Olympus BX53 optical microscope (Olympus Corporation, Tokyo, Japan), the fine fraction was found to be the most homogeneous and hydrated and also demonstrated a relatively high initial sorption rate for poorly soluble organic compounds (Figure 2). The medium fraction showed signs of drying despite having the same amount of water and hydrogel concentration as the fine fraction. At the same time, the medium fraction was the most effective at retaining the organic compound on the particle surface (Figure 3), which was visualized as a dark, opaque layer on the surface. The coarse-grained fraction was the least hydrated, dried faster than the other fractions, and demonstrated lower efficiency in retaining the organic compound (Figure 4).

**Figure 5:**
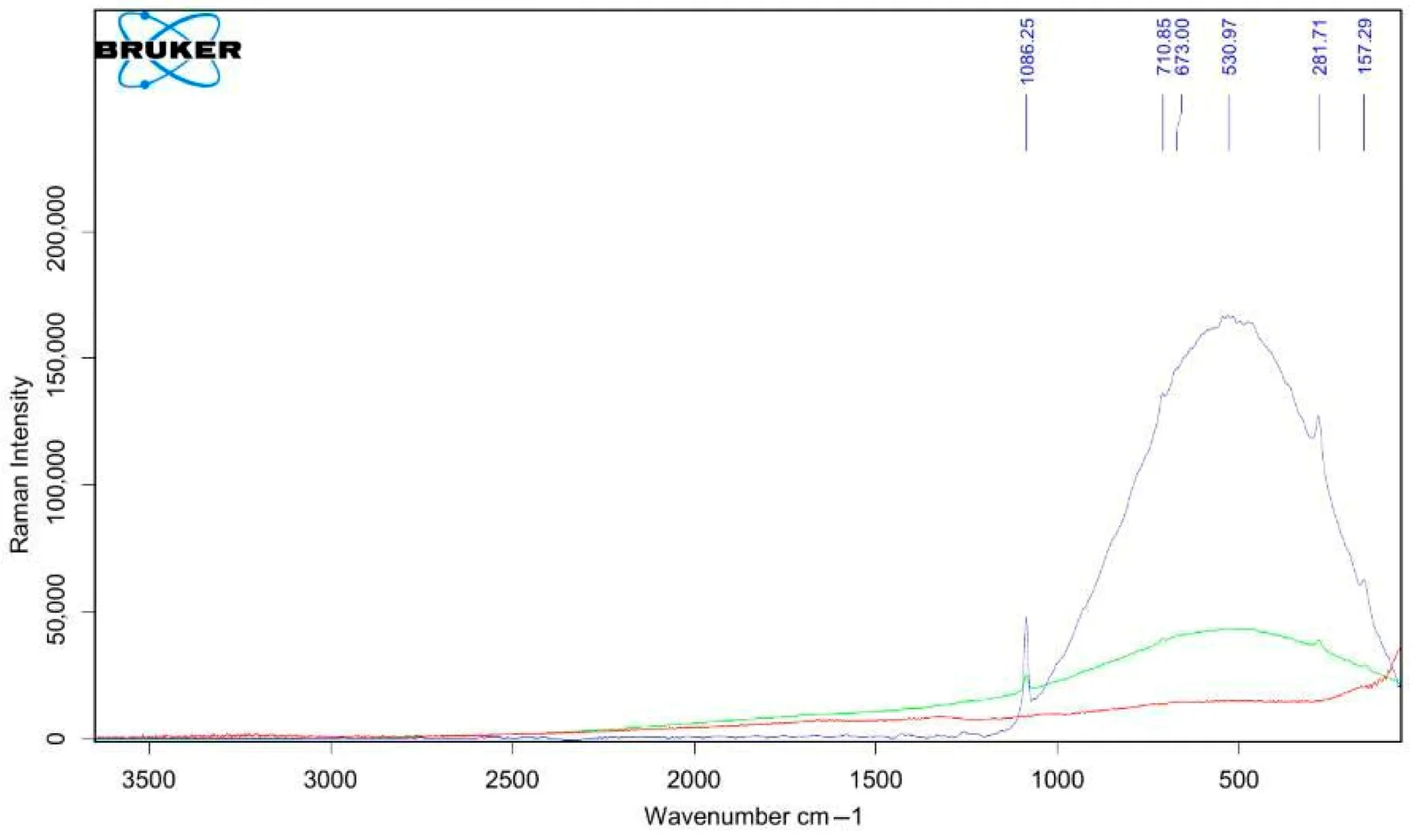
Results of spectral analysis of the hydrogel.

A Senterra II confocal Raman microscope (Bruker Optics GmbH, Ettlingen, Germany) was used for the measurements. A laser with a wavelength of 785 nm and a power of 50 mW was used, with an acquisition time of 10 s; data were collected in triplicate (Figure 5). Raman spectra were processed using OPUS software (Bruker Optics GmbH, Ettlingen, Germany; version 9.3).

Raman spectra were analyzed in the 100–3500 cm^−1^ range. Peak positions were determined from the averaged spectra of three independent measurements for each fraction. The spectra were baseline-corrected and normalized to the intensity of the 1086 cm^−1^ band to allow comparison between fractions. Reference Raman spectra were recorded under the same conditions for pure thiamine, succinic acid, heteroauxin, and the pristine hydrogel without adsorbed stimulants. The intensity ratios of the characteristic bands at 710, 673, 531, 282, and 157 cm^−1^ relative to the 1086 cm^−1^ band were calculated for each hydrogel fraction and compared with the corresponding ratios in the reference spectra.

**Figure 6:**
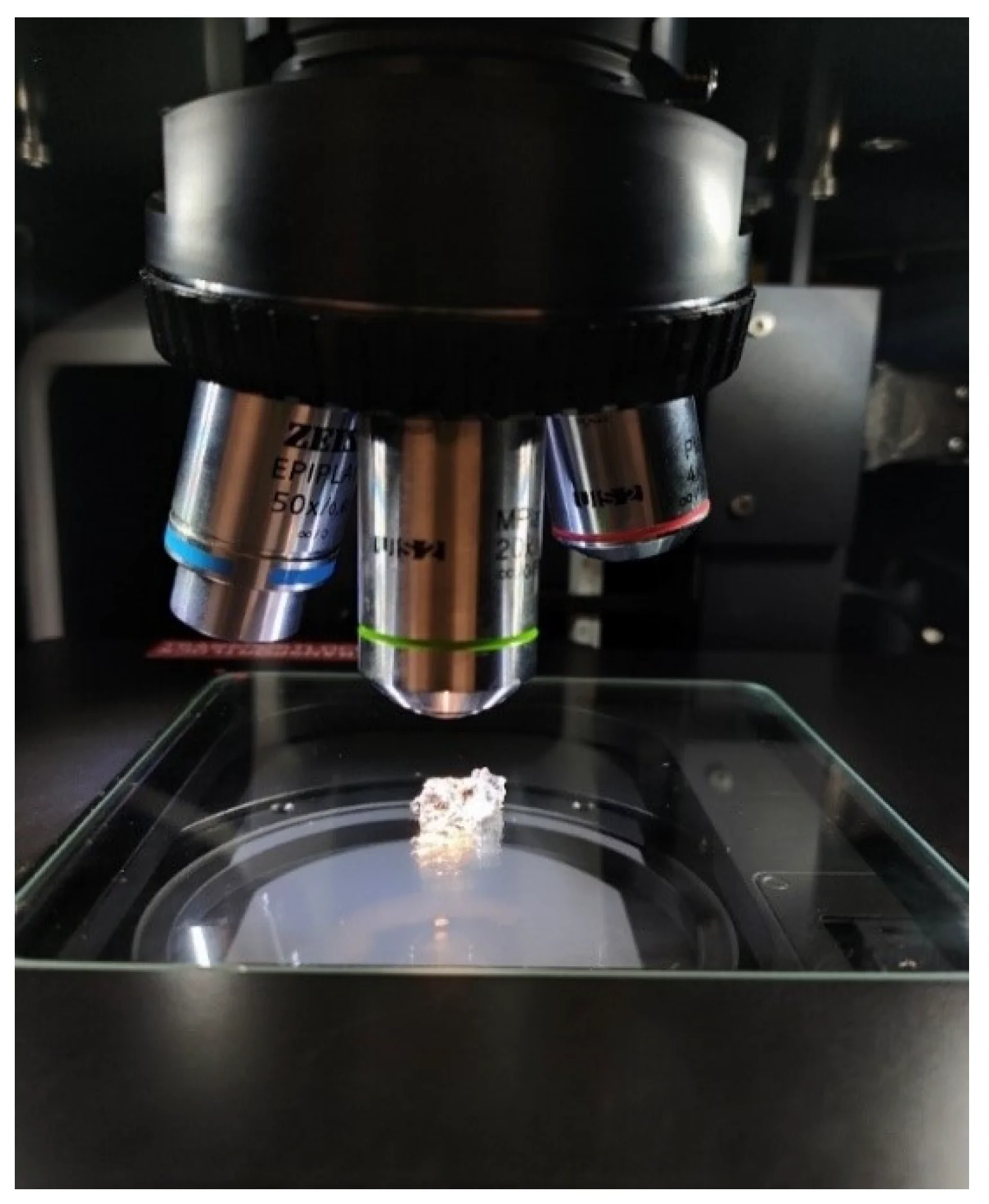
The process of optical analysis of the hydrogel.

The differences between the averaged spectra obtained were substantial. The medium-fraction hydrogel demonstrated a more pronounced spectrum of organic compounds, including amide and hydrocarbon peaks. The coarse-grained hydrogel did not exhibit a clearly defined spectrum in the 600–1100 cm^−1^ region, except for a characteristic increase in intensity in the low-frequency range (0–50 cm^−1^). The fine-grained hydrogel exhibited peaks characteristic of the organic compound, although they were less pronounced (Figure 6).

#### 2.1.2. Preparation of the Composite Polymeric Medium

4.0 g of dry hydrogel was weighed for hydration and poured into 100 mL of distilled or deionized water at 20 ± 2 °C. Water with hardness ions (Ca^2+^, Mg^2+^) above 2 mmol/L was not used, as they reduce the water absorption of potassium hydrogels. The hydrogel was added gradually with continuous stirring for 1–2 min, after which it was left to swell in a dark place at a temperature of 20 ± 1 °C for 60 min, during which time more than 95% of the maximum volume was reached.

After the swelling was completed, the excess free water was carefully removed through a 0.5 mm mesh sieve. As a result, elastic, flexible granules with a diameter of 3–8 mm were obtained without surface moisture [29,30]. To prepare a composite polymeric medium, 160 g of vermicompost sifted through a 2 mm sieve and 80 g of perlite (2–5 mm fraction) were added to the hydrated gel. The dry weight ratio was hydrogel: vermicompost: perlite = 1: 40: 20. Mixing was carried out in two stages: 2 min in a rotary mixer (DLAB MX-RL-E; DLAB Scientific Co., Ltd., Beijing, China) at low speed, then 1 min manually with a spatula to homogenize without destroying the gel granules. The resulting mixture had a volume density of 0.55–0.65 g/cm3, an aeration threshold of 22–25% and a moisture capacity of 65–70% of the total moisture capacity.

An optimized commercial substrate was used for the control variant: peat (Klasmann TS3), perlite (2–5 mm) and vermicompost in a volume ratio of 50:30:20. Target parameters: pH 6.2 ± 0.1; EC 1.2 ± 0.1 mS/cm.

For the pure hydrogel version, the hydrated gel was used without additives.

The resulting composite polymeric medium is a homogeneous porous mass with a volume of approximately 580–600 mL, with a moisture content of 65–70% of the total moisture capacity, a density of 0.65–0.75 g/cm3 and a pH of 5.8–6.5; when compressed in the palm of the hand, the lump retains its shape for 2–3 s, after which it crumbles without releasing free water. The medium is distributed over sterile plastic cups for growing plants with a volume of 250 mL (diameter 70 mm, height 70 mm), filling them to a level of 5 cm from the top (about 200 mL per cup), after which they are slightly tamped with a finger and pre-moistened with 15 mL of distilled water at a temperature of 20 °C, allowing it to soak within 15 min. The prepared samples were kept in the dark at 20 ± 1 °C for 24 h to stabilize the physicochemical parameters (uniform distribution of K^+^ ions, adaptation of the microbial community). After that, the moisture content was checked: the target value was 70 ± 5% HB, which corresponded to a mass of the filled cylinder of 279 ± 5 g (dry component weight 164 g + water 115 g). Strawberry rosettes with 3–4 leaves and roots at least 2 cm long, without signs of damage, were selected as standard; Before planting, the roots were washed for 1 min in 0.01% potassium permanganate solution and then rinsed twice with distilled water for 30 s each.

The moisture content should not fall below 50% HB (inhibition of root formation) or exceed 80% HB (risk of anaerobiosis and rotting of the root collar). At the end of the experiment (on days 7, 14, and 21), the rooting percentage, main root length, number of adventitious roots, fresh and dry mass of the extract (1:5), and potassium ion concentration (flame photometry; A. Krüss Optronic FP8400, A. Krüss Optronic GmbH, Hamburg, Germany)were recorded to monitor the release of macronutrients from the hydrogel.

To ensure reproducibility, all operations were performed at the same time of day (preferably between 9:00 and 11:00 a.m.), one batch of hydrogel and vermicompost was used throughout the experiment, and an optimized substrate (peat: perlite: vermicompost = 50:30:20), moistened to 70% HB, was used as the control. If necessary, a phytohormone (e.g., indole butyric acid, 50 mg/L) could be added to individual variants; however, this requires a separate assessment of its compatibility with the polymer matrix.

**Figure 7:**
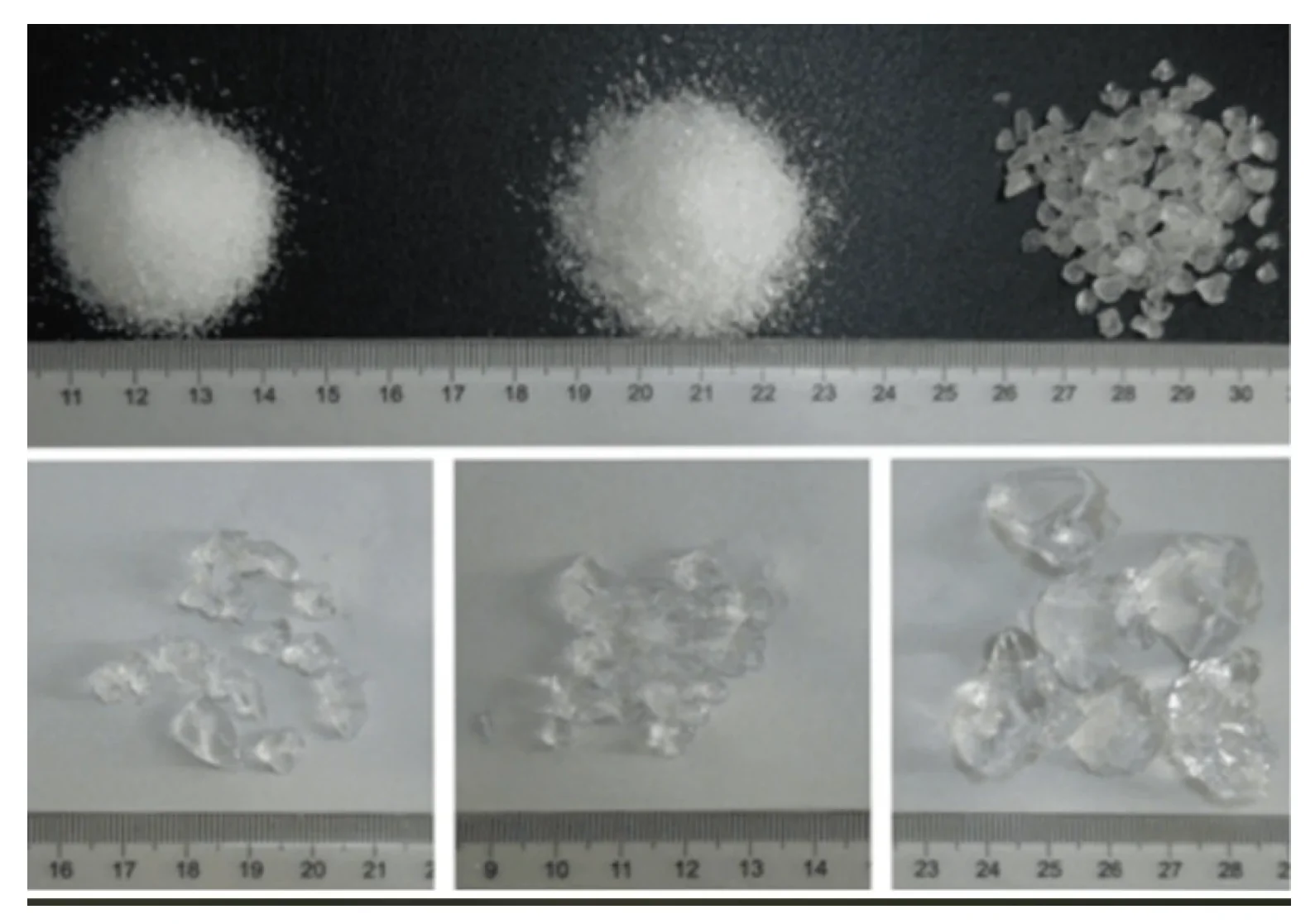
Selection of potassium hydrogel fractions.

The experimental procedure for assessing the effect of composite polymeric media on the rooting and initial growth of garden strawberry rosettes (*Fragaria* × *ananassa*) was conducted in a climate chamber according to the described methodology. Dry, pre-fractionated potassium polyacrylate hydrogel (B 415 K) was used to prepare the experimental composite polymeric medium. The medium fraction (0.5–1.5 mm) was selected based on preliminary spectroscopic studies, as it demonstrated the best balance between hydration rate and structural integrity (Figure 7). Vermicompost was used as the organic component, while perlite with a particle size of 2–5 mm was added to improve aeration. The vermicompost underwent laboratory testing: moisture content was 28%, pH of the aqueous extract was 6.8, humic acid content was 2.9%, and no viable weed seeds or pathogenic microflora were detected.

The medium preparation procedure was described and standardized in detail. A mass of 4.00 ± 0.05 g of dry hydrogel was weighed on an analytical balance (Sartorius Entris II BCE1241S; Sartorius AG, Göttingen, Germany) and gradually added to 100 mL of water (temperature 20 °C, resistivity 18.2 mω · cm) with constant mechanical stirring at a speed of 300 rpm. After initial dispersion for 2 min, the mixture was left to swell completely for 60 min in the dark at 20 ± 0.5 °C.

**Figure 8:**
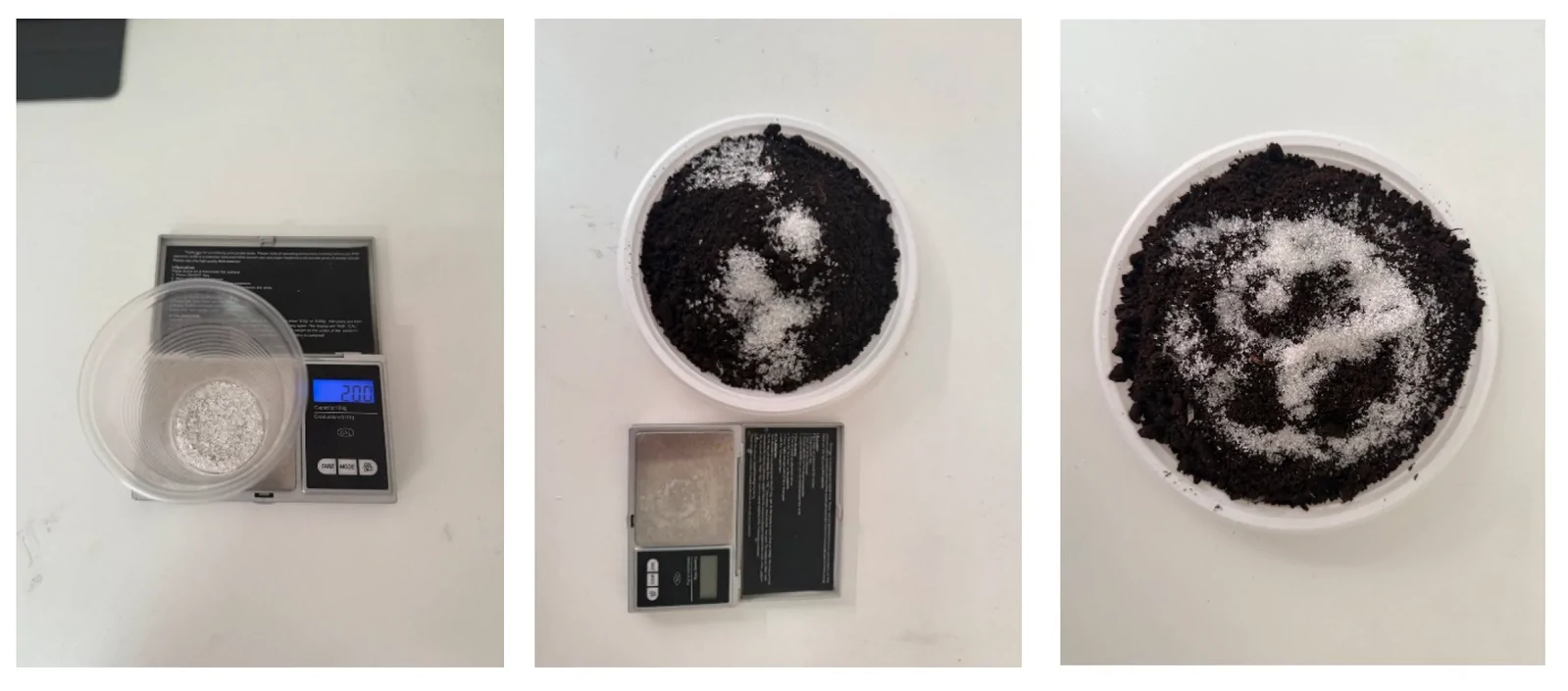
The process of preparing a composite polymeric medium based on hydrogel and vermicompost.

After this period, the final volume of the hydrated gel was recorded, and excess free water was removed by vacuum filtration through a sieve with a pore size of 0.5 mm. Then, 160.0 ± 0.5 g of vermicompost, sieved through a 2-mm mesh, was added to the resulting elastic gel mass. Mixing was performed in two stages: first, for 2 min in a rotary mixer at low speed for preliminary distribution, followed by 1 min of manual mixing with a spatula for final homogenization without destroying the gel granules. The control variant (C) was prepared using the optimized substrate and adjusted to the same moisture level of 70% HB by adding a calculated volume of deionized water. In parallel, a second experimental variant based on pure hydrated hydrogel without vermicompost was prepared (Figure 8).

**Figure 9:**
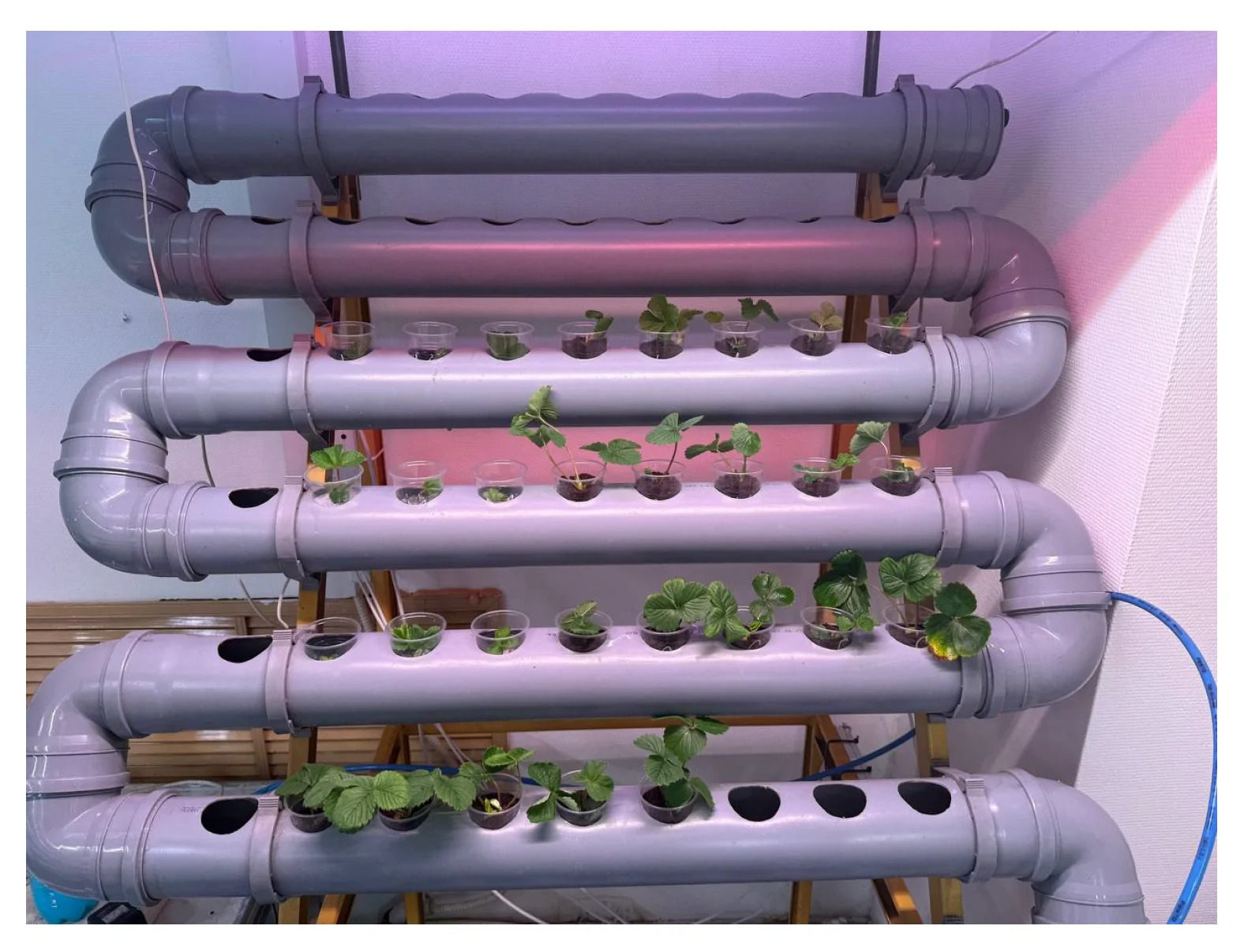
The strawberry planting processes.

**Figure 10:**
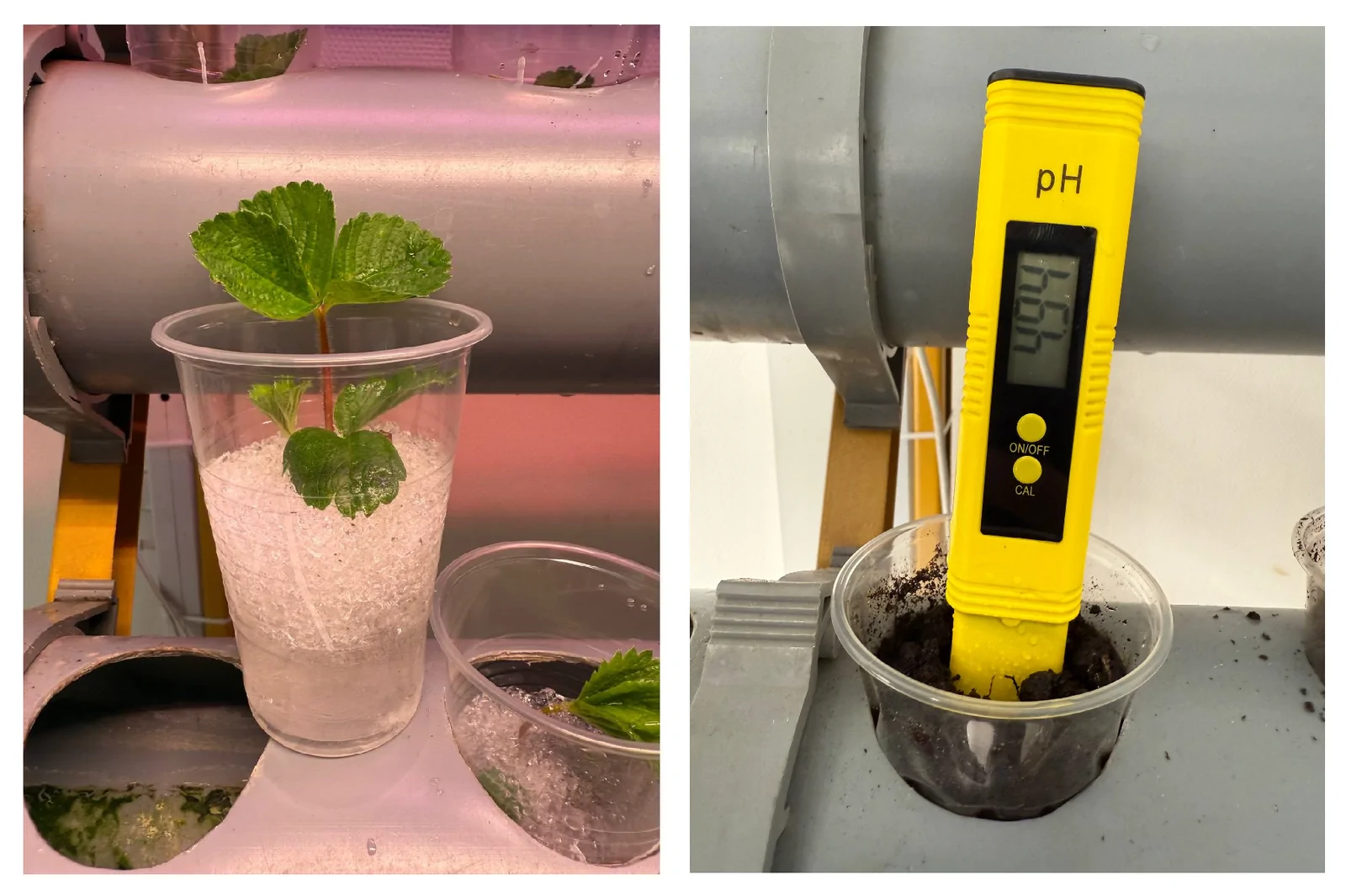
The process of planting plants to study the effect of the composite polymeric medium on strawberry rooting.

The prepared substrates were used to fill 135 perforated plastic cups with a volume of 250 mL (45 cups for each experimental variant). One calibrated rosette of the ‘Alba’ strawberry cultivar, with three fully developed leaves and a main root length of 2.5 ± 0.3 cm, was planted in each cup (Figure 9). Before planting, the root system was treated with a 0.01% KMnO_3_ solution and subsequently rinsed. The plants were grown in a phytotron at a temperature of 20/18 °C (day/night), relative air humidity of 75 ± 5%, and a 16-h photoperiod with a photosynthetically active radiation (PAR) intensity of 250 µmol/m^2^·s (Figure 10).

#### 2.1.3. Swelling Ratio and Elemental Analysis

The swelling ratio of each hydrogel fraction was determined as the ratio of the mass of the swollen gel, after removal of free water, to the mass of the dry hydrogel. Measurements were performed in triplicate for each fraction, and the resulting values were averaged. Structural stability of the swollen granules was assessed quantitatively as the fraction of intact granules after 24 h of swelling in distilled water, determined by optical microscopy on three independent samples of 100 granules per fraction. A granule was considered intact if it retained its shape and did not disintegrate into fragments.

Inductively coupled plasma optical emission spectrometry (ICP OES Thermo iCap 6300duo, Thermo Fisher Scientific, Waltham, MA, USA) was used to determine the concentration of Na, K, Ca, and Mg ions in the control solution and in experimental solutions passed through a hydrogel.

It should be noted that the data obtained and presented below reflect the concentrations of passed solutions, not hydrogel, and that part of the solution was retained by the gel during passage through the hydrogel. To ensure comparable conditions, 5 mL aliquots of solutions passed through a hydrogel were taken, diluted 100 times, and measured using the method described above (Table 1).

To quantify the adsorption capacity of the hydrogel fractions for organic growth stimulants, batch adsorption experiments were carried out using thiamine and succinic acid as single-component model adsorbates. Dry hydrogel fractions (0.100 ± 0.005 g) were hydrated in 50 mL distilled water for 60 min at 20 ± 1 °C. The hydrated gel was then transferred into 50 mL of thiamine or succinic acid solution with initial concentrations of 10–200 mg/L at pH 6.0. The suspensions were shaken at 100 rpm and 20 ± 1 °C. Aliquots were withdrawn at 0, 5, 10, 20, 30, 60, 120, 240, 360, 480, 720, and 1440 min, filtered through a 0.45 μm membrane, and analyzed. Thiamine was determined by UV–Vis spectrophotometry (Shimadzu UV-1900i Plus; Shimadzu Corporation, Kyoto, Japan) at 245 nm, and succinic acid was determined by HPLC with UV detection (Agilent 1260 Infinity II; Agilent Technologies, Santa Clara, CA, USA) at 210 nm. The adsorption capacity at time t and at equilibrium was calculated from the measured decrease in adsorbate concentration in solution, taking into account the solution volume and the dry mass of the hydrogel. Kinetic data were fitted to pseudo-first-order and pseudo-second-order models, and equilibrium data were fitted to Langmuir and Freundlich isotherms. All experiments were performed in triplicate.

### 2.2. Field Experiment: Method of Applying the Composite Medium

The field experiment was designed as a validation step for the composite polymeric medium, with the primary aim of confirming that its water-retention and ion-exchange functions are preserved under open-ground conditions. The agronomic parameters (survival rate, root length, biomass) are reported as indicators of the medium performance, not as the primary endpoint of the study.

To evaluate the effectiveness of the developed composite medium under field conditions, a field experiment was conducted. Unlike the laboratory experiment, in which the plants were grown in plastic containers under controlled microclimatic conditions, the field trial was carried out in open ground under natural light and temperature conditions. The control consisted of unmodified soil (sod-podzolic loam, pH 5.8–6.2, humus content 2.1–2.4%) without the application of any water-retaining additives.

It should be emphasized that the control variants in the laboratory and field experiments were deliberately different because the two experiments addressed different objectives. In the laboratory experiment, the aim was to compare the developed composite polymeric medium with the optimized commercial substrate (peat:perlite:vermicompost = 50:30:20) that is currently used in protected cultivation and nursery production, i.e., against the best available substrate under controlled conditions. In the field experiment, the aim was to validate the practical applicability of the composite medium under open-ground conditions, where the actual farming practice is planting into unamended natural soil without any water-retaining additives. Therefore, the field control was unamended sod-podzolic loam, which represents the real baseline for open-ground strawberry cultivation in the region. This difference in controls reflects the different purposes of the two experiments rather than an inconsistency in the experimental design: the laboratory experiment tests the composite medium against an optimized substrate, whereas the field experiment tests it against current agricultural practice.

**Figure 11:**
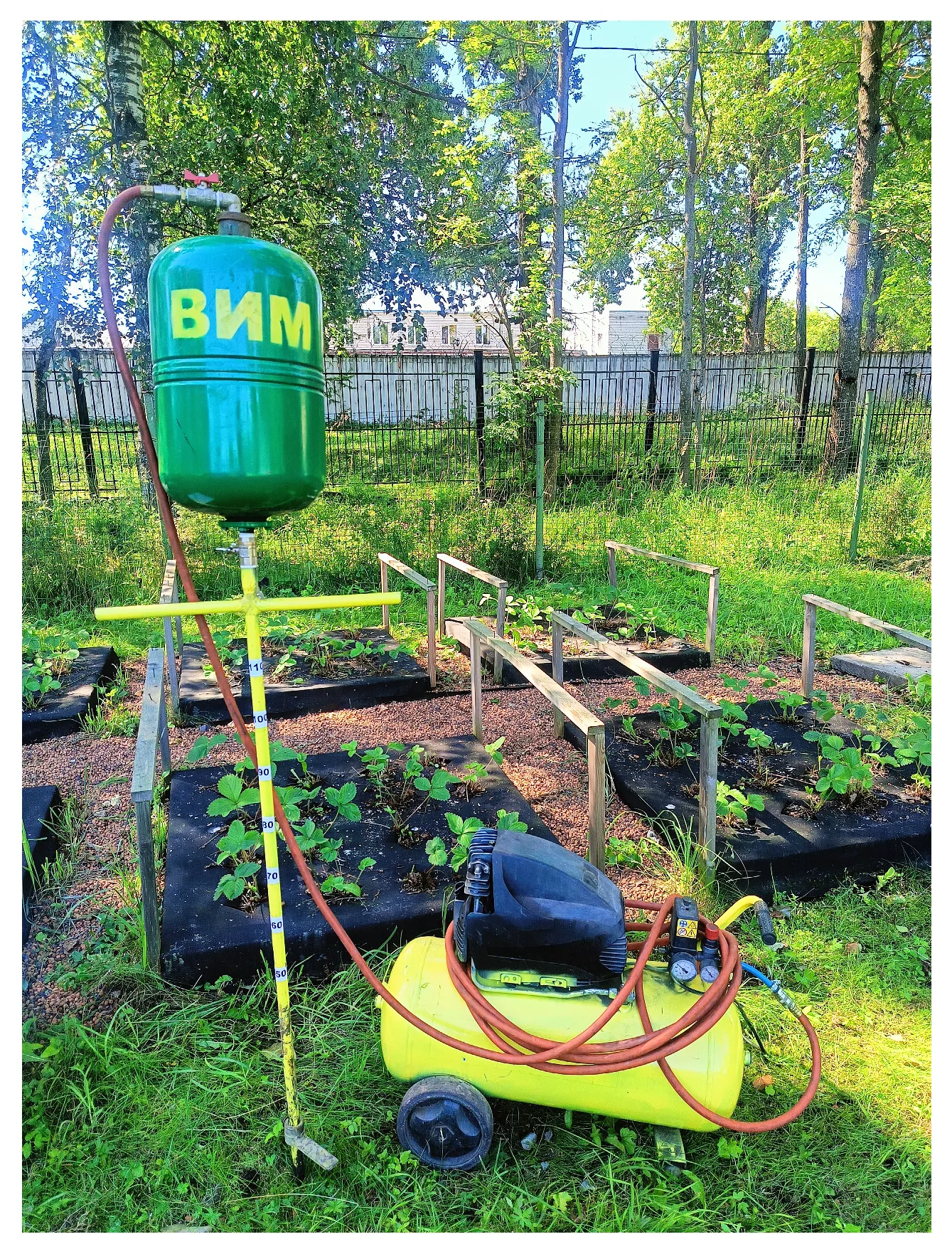
Manual auger for root-zone application of the composite hydrogel medium under field conditions.

The composite medium, consisting of medium-fraction potassium polyacrylate hydrogel (0.5–1.5 mm), vermicompost, and perlite at a dry-component ratio of 1:40:20, was prepared according to the procedure described in Section 2.1. The hydrogel was hydrated immediately before planting using deionized water at 20 ± 2 °C, at a rate of 4 g of dry polymer per 100 mL of water. A manual soil auger was used to apply the prepared composite medium to the root zone. The tool enabled vertical channels 3.5 cm in diameter and up to 30 cm deep to be formed in the soil without significant disturbance of the upper soil layer (Figure 11).

The application procedure consisted of three consecutive operations: (1) formation of a planting hole in the root zone using the soil auger; (2) measured application of the composite medium (approximately 50 mL per plant) directly into the root zone; and (3) backfilling with soil followed by light compaction. In the control variant, planting was carried out in the same manner, but without the application of the composite medium.

The experimental design included three replicates, with 20 ‘Alba’ strawberry plants per plot (180 plants in total). Rosettes with 3–4 leaves and a root length of 2.5 ± 0.3 cm were planted with a row spacing of 30 cm and an in-row spacing of 25 cm. Irrigation was performed as needed when soil moisture decreased to 55–60% HB, at a rate of 5 L/m^2^. In the experimental variant, irrigation was performed less frequently, when soil moisture decreased to 45–50%, corresponding to a 30% reduction in irrigation frequency.

The functional performance of the polymeric medium was assessed by monitoring soil moisture in the root zone (thermogravimetric method) and exchangeable K^+^ concentration in the soil solution (flame photometry), which reflect the water-retention and ion-exchange functions of the hydrogel. In parallel, plant establishment (%), root system morphometry, biomass, and chlorophyll content were recorded as indicators of the medium performance under field conditions. The effectiveness of the treatment was assessed on days 7, 14, and 21 after planting using the following parameters: plant establishment (%); main root length and total root system length (determined by scanning followed by image analysis using ImageJ 1.54t); number of third- and fourth-order lateral roots; fresh and dry biomass of roots and aboveground parts (after drying at 105 °C to constant weight); and leaf chlorophyll a and b content (determined spectrophotometrically after extraction with 96% ethanol). Plants were assigned to treatments in a randomized complete block design. Biological replicates are defined as individual plants (*n* = 60 per treatment for field measurements), and technical replicates are defined as independent measurements on the same sample (*n* = 3). All measurements were performed on coded samples to avoid observer bias. Data are presented as mean ± standard error (SE), and error bars in all figures represent SE. Statistical analysis was performed using one-way ANOVA followed by Tukey’s post hoc test at *p* < 0.05; normality and homogeneity of variances were confirmed before analysis. It should be noted that the functional performance of the composite medium was assessed during the initial 21-day rooting stage only; the degradation behaviour and the retention of water-retention and K^+^-release functions after repeated cultivation cycles were not evaluated in the present study and are considered a direction for future work.

## 3. Results

### 3.1. Fraction-Dependent Properties of the Hydrogel

Raman analysis. The band at 1086 cm^−1^ is assigned to C–O and C–C stretching vibrations of the immobilized organic growth stimulant and to skeletal vibrations of the polyacrylate network, indicating effective sorption of heteroauxin on the polymer surface. The bands at 710 and 673 cm^−1^ correspond to C–H out-of-plane deformation vibrations of the aromatic ring. The band at 531 cm^−1^ is attributed to symmetric stretching of carboxylate groups (COO^−^) of the polyacrylate network. The low-frequency bands at 282 and 157 cm^−1^ are assigned to skeletal vibrations of the polymer backbone and lattice modes, respectively. The medium fraction demonstrated the most pronounced spectrum of the immobilized organic compounds, whereas the coarse fraction did not exhibit clearly defined bands in the 600–1100 cm^−1^ region, indicating lower sorption capacity. Quantitative comparison of the Raman spectra with the reference spectra confirmed the immobilization of the growth stimulants on the hydrogel surface. The reference spectrum of pristine hydrogel showed only the carboxylate band at 531 cm^−1^ and the skeletal bands at 282 and 157 cm^−1^, whereas the bands at 710 and 673 cm^−1^ were absent, confirming that these bands originate from the aromatic ring of the adsorbed stimulants rather than from the polyacrylate network. The intensity ratios I_710_/I_1086_ and I_673_/I_1086_ were highest for the medium fraction (0.42 ± 0.03 and 0.38 ± 0.02, respectively), intermediate for the fine fraction (0.28 ± 0.02 and 0.25 ± 0.02), and lowest for the coarse fraction (0.11 ± 0.01 and 0.09 ± 0.01), with statistically significant differences (*p* < 0.05). These ratios correlate with the equilibrium adsorption capacities determined by batch adsorption experiments, providing independent quantitative confirmation that the medium fraction exhibits the highest sorption of organic growth stimulants. These results confirm that the medium fraction provides the optimal balance between hydration, structural integrity, and sorption of bioactive compounds.

Quantitative batch adsorption confirmed the fraction-dependent ranking observed by Raman spectroscopy. The medium fraction exhibited the highest equilibrium adsorption capacities for both thiamine and succinic acid. For thiamine, the equilibrium adsorption capacity was 42.5 ± 2.1 mg/g for the medium fraction, 31.8 ± 1.7 mg/g for the fine fraction, and 18.6 ± 1.2 mg/g for the coarse fraction. For succinic acid, the corresponding values were 38.2 ± 1.9 mg/g, 29.4 ± 1.5 mg/g, and 16.9 ± 1.1 mg/g, respectively. The medium fraction thus exceeded the fine fraction by 25–30% and the coarse fraction by 2.1–2.3 times. The adsorption kinetics were best described by the pseudo-second-order model with R^2^ = 0.986–0.995, indicating that the adsorption process is governed by chemical interactions with carboxylate groups of the polyacrylate network rather than by pure physical diffusion. The equilibrium data were well fitted by the Langmuir isotherm with R^2^ = 0.978–0.991. The maximum monolayer adsorption capacities for the medium fraction were 46.8 mg/g for thiamine and 41.5 mg/g for succinic acid. The fine fraction showed a faster initial uptake but a lower equilibrium capacity, which is attributed to its higher swelling ratio and looser network structure, whereas the coarse fraction exhibited slower diffusion and lower surface area. These quantitative results provide direct evidence that the medium fraction of 0.5–1.5 mm has the optimal adsorption performance for organic growth stimulants, in agreement with its most pronounced Raman bands at 1086, 710, 673, 531, 282, and 157 cm^−1^.

Swelling behavior. The medium hydrogel fraction demonstrated a swelling ratio of Q = 19.1 ± 0.6 g/g, compared with 23.4 ± 0.8 g/g for the fine fraction and 12.7 ± 0.9 g/g for the coarse fraction. The higher swelling ratio of the fine fraction is attributed to its larger specific surface area and faster water penetration into the polymer network, whereas the coarse fraction exhibited lower hydration due to reduced surface-to-volume ratio and slower diffusion of water into the granule core. The medium fraction showed an intermediate swelling ratio but the highest structural integrity after swelling and the most effective immobilization of organic growth stimulants. These results indicate that the swelling behavior of the hydrogel fractions is governed by the balance between surface area, cross-linking density, and structural stability of the granules. Structural stability followed the order: medium (94.2 ± 2.1% intact granules) > coarse (88.5 ± 2.6%) > fine (79.6 ± 3.2%), with statistically significant differences (*p* < 0.05). The lower stability of the fine fraction is attributed to its higher swelling ratio and looser network structure, whereas the coarse fraction showed reduced swelling but moderate stability. Taken together, the three quantitative parameters—swelling ratio (fine 23.4 ± 0.8, medium 19.1 ± 0.6, coarse 12.7 ± 0.9 g/g), structural stability (fine 79.6 ± 3.2, medium 94.2 ± 2.1, coarse 88.5 ± 2.6%), and sorption capacity (thiamine: fine 31.8 ± 1.7, medium 42.5 ± 2.1, coarse 18.6 ± 1.2 mg/g; succinic acid: fine 29.4 ± 1.5, medium 38.2 ± 1.9, coarse 16.9 ± 1.1 mg/g)—demonstrate that only the medium fraction combines high swelling, the highest structural stability, and the highest sorption capacity, justifying its selection as the optimal fraction for the composite polymeric medium. This is consistent with the ICP-OES data (Table 1), which show that the solutions passed through the hydrogel were depleted in K^+^, Ca^2+^, and Mg^2+^ relative to the initial Hoagland solution, indicating that the polyacrylate network interacts with multivalent cations and that the swelling capacity measured in distilled water represents an upper estimate of the swelling behavior under rhizosphere conditions.

**Figure 12:**
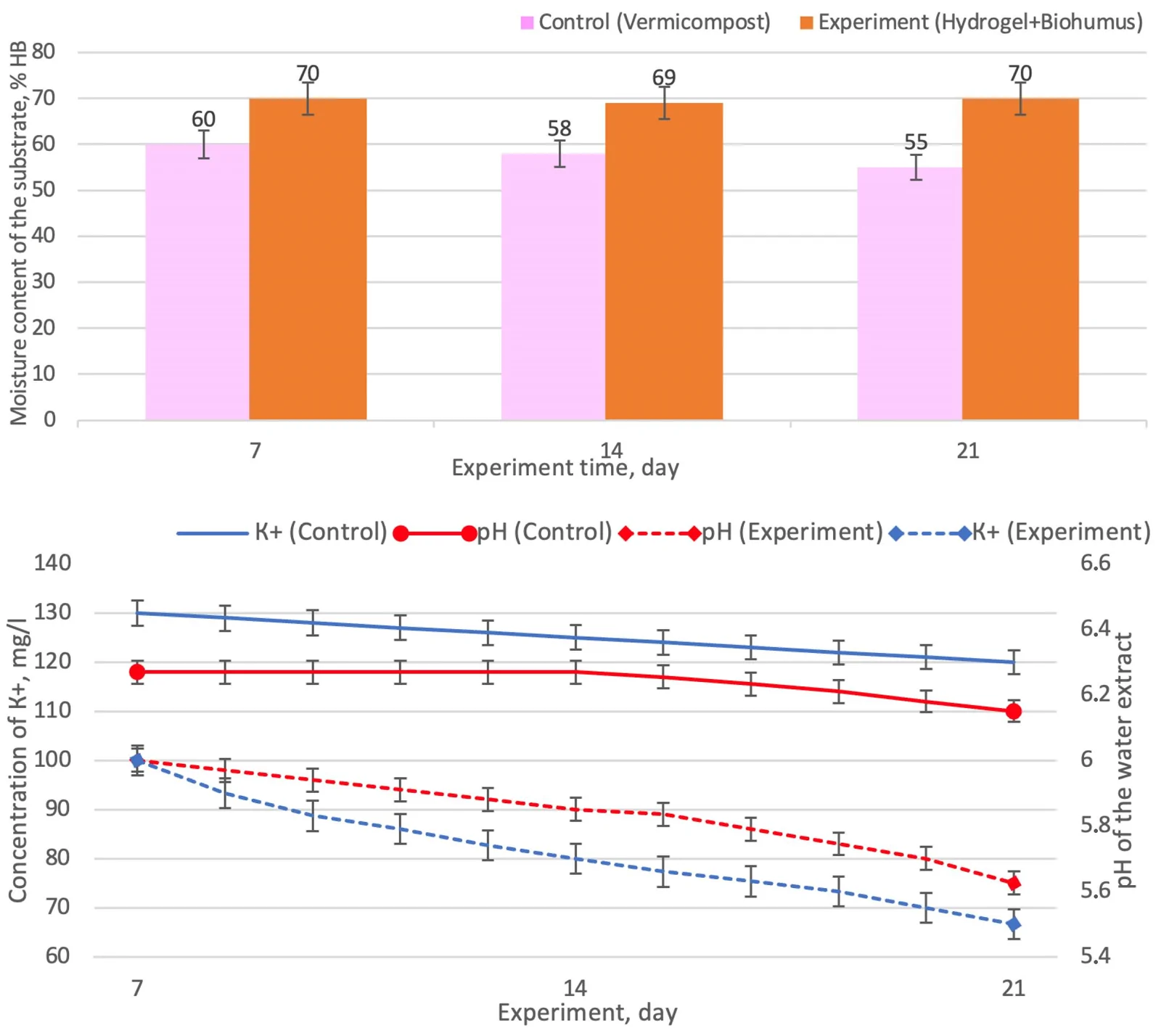
Dynamics of agrophysical parameters of the substrate during the strawberry rooting experiment.

The graph presents changes in moisture content, available potassium (K^+^) concentration, and pH of the aqueous extract in the control and experimental variants over the 21-day experimental period (Figure 12).

### 3.2. Water Retention and Ion-Exchange Behavior of the Composite Medium

Water retention. The dynamics of moisture content in the composite polymeric medium, pure hydrogel, and control substrate over 21 days are shown in Figure 12. The composite medium maintained moisture within 68–72% of full water-holding capacity throughout the experiment, whereas the control decreased to 55–60% between irrigations. The water retention curve of the composite medium indicates that the hydrogel matrix acts as a buffering reservoir, reducing the amplitude of moisture fluctuations. The pure hydrogel maintained moisture at 70–75% HB, but its structural stability was lower than that of the composite medium. The difference between the composite medium and the control was most pronounced between days 7 and 14, when the control substrate reached its minimum moisture content after irrigation.

Nutrient dynamics and potassium release. The concentration of exchangeable K^+^ in the extract from the composite medium remained stable at 120–135 mg/L over 21 days, whereas in the control it decreased linearly from 100 to 65 mg/L. This release profile indicates that the polyacrylate network functions as an ion-exchange reservoir, sustaining K^+^ availability without additional mineral fertilization. The K^+^ release curve of the composite medium shows only minor fluctuations, which is consistent with the ion-exchange mechanism controlled by carboxylate groups of the polymer network. In contrast, the monotonic decrease in K^+^ in the control reflects progressive depletion of available potassium due to leaching and plant uptake. It should be noted that vermicompost also contains exchangeable K^+^ and therefore contributes to the total K^+^ pool, but since the same amount of vermicompost was present in both the composite medium and the control, and the control nevertheless showed a monotonic decrease in K^+^, the stability of K^+^ in the composite medium cannot be attributed to vermicompost alone.

In the pure hydrogel variant, the K^+^ concentration was 90–110 mg/L. Under the experimental conditions, pH values remained within the range optimal for strawberries (6.1–6.3), whereas vermicompost showed a gradual shift toward acidity, reaching 5.7. In the pure hydrogel, pH was maintained at 6.0–6.2.

### 3.3. Demonstration of Functional Performance in a Model Root-Zone Application

**Figure 13:**
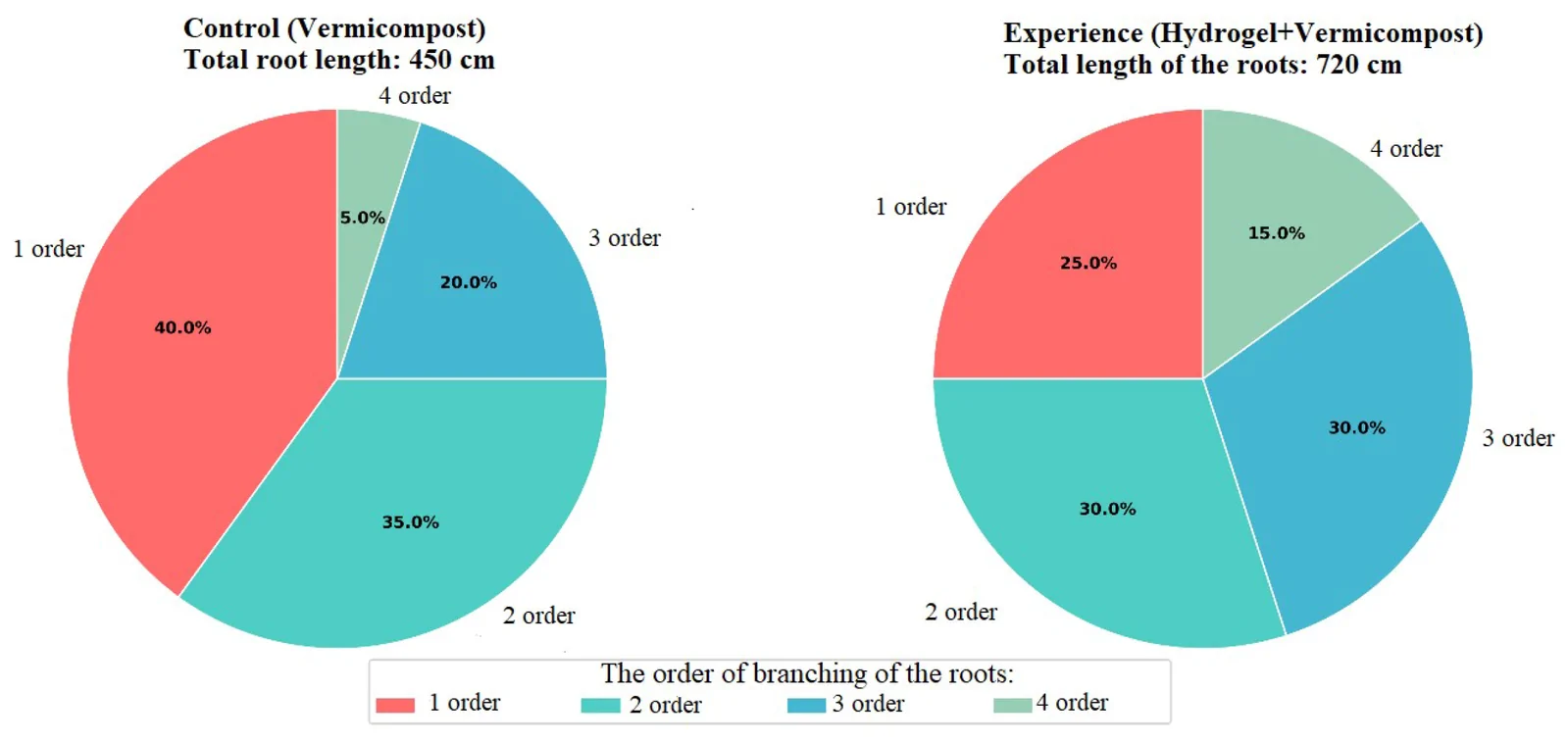
Comparative characteristics of the strawberry root system on the 21st day of rooting.

The condition of the medium and plants was monitored on days 7, 14, and 21. At each stage, 15 plants from each group were selected for analysis (Figure 13). A number of morphophysiological parameters were measured:

1. Rooting percentage—the proportion of plants actively forming new roots.
2. Root system parameters: main root length, total root length (using scanning and image analysis), number of roots of the first to fourth orders, and fresh and dry biomass of roots and aboveground parts (after drying at 105 °C to constant weight).
3. Substrate condition: pH and electrical conductivity of the aqueous extract (1:5), actual moisture content determined by the thermogravimetric method, and available potassium concentration in the extract determined by flame photometry.
4. Physiological state of plants: chlorophyll *a* and *b* content in the leaves determined spectrophotometrically after extraction with 96% ethanol, and visual assessment using a 5-point scale (leaf blade coloration and signs of chlorosis or necrosis).

The pie charts show the distribution of total root length according to branching order in the control and experimental variants.

**Figure 14:**
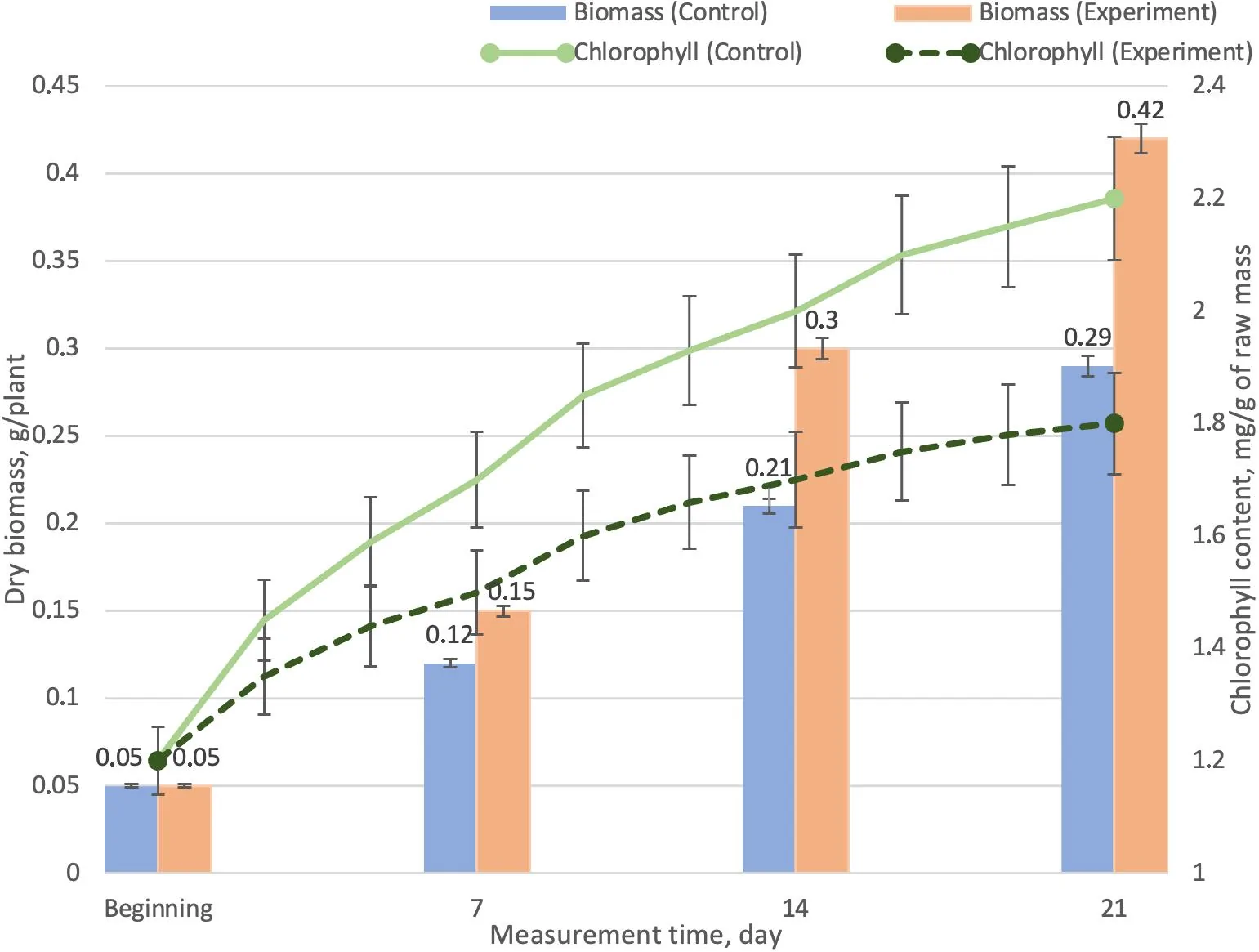
Dynamics of biomass accumulation and chlorophyll content in strawberry rosettes during rooting.

The dynamics of observations revealed statistically significant differences between the experimental variants as early as the seventh day. In the combined medium (hydrogel + vermicompost + perlite), rooting reached 100%, whereas in the control it was 86%. The main factor was the stability of the water regime: moisture content in the experimental variant fluctuated within a narrow range of 68–72% HB, whereas in pure vermicompost it decreased to 55–60% between irrigations, causing short-term water stress in the plants. In the composite medium, optimal values of redox potential (Eh), ranging from +150 to +220 mV, and dissolved oxygen (O_2_) concentration, ranging from 5.2 to 6.5 mg/L, were maintained. In the control, by day 14, Eh decreased to +80 to +120 mV and O_2_ concentration decreased to 3.8–4.5 mg/L, indicating temporary hypoxia. Electrical conductivity (EC) in the composite medium remained stable at 1.15–1.25 mS/cm, whereas in the control it decreased from 1.20 to 0.85 mS/cm by day 21. By day 21, the advantage of the combined medium was evident across all parameters (Figure 14).

Root morphometry. The mean main root length in the experimental variant was 15.8 ± 1.2 cm, compared with 11.2 ± 1.5 cm in the control. The total root system length exceeded the control values by 1.6-fold, while the number of third- and fourth-order lateral roots was 1.8–2.1 times higher. Root dry biomass in the experimental variant was 0.42 ± 0.03 g per plant, which was 45% higher than in the control (0.29 ± 0.04 g per plant). The second experimental variant (pure hydrogel) showed intermediate results: rooting—92%, main root length—13.5 ± 1.3 cm, and root biomass—0.35 ± 0.03 g per plant.

**Figure 15:**
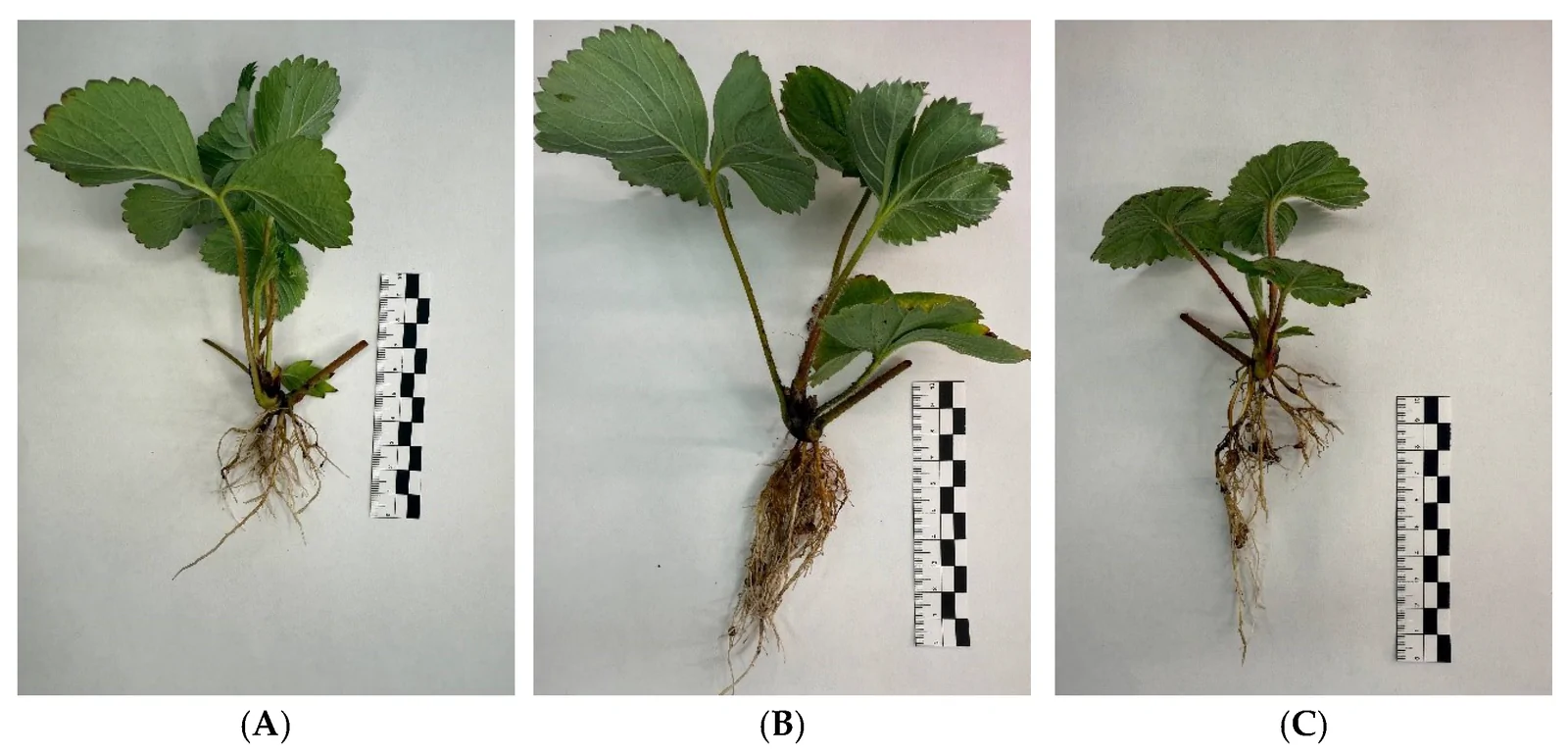
(**A**)—control; (**B**)—pure hydrogel; (**C**)—composite polymeric medium. Assessment of morphometry of strawberry roots.

Plants grown on pure hydrogel exhibited less pronounced signs of stress compared with the control group (Figure 15).

**Figure 16:**
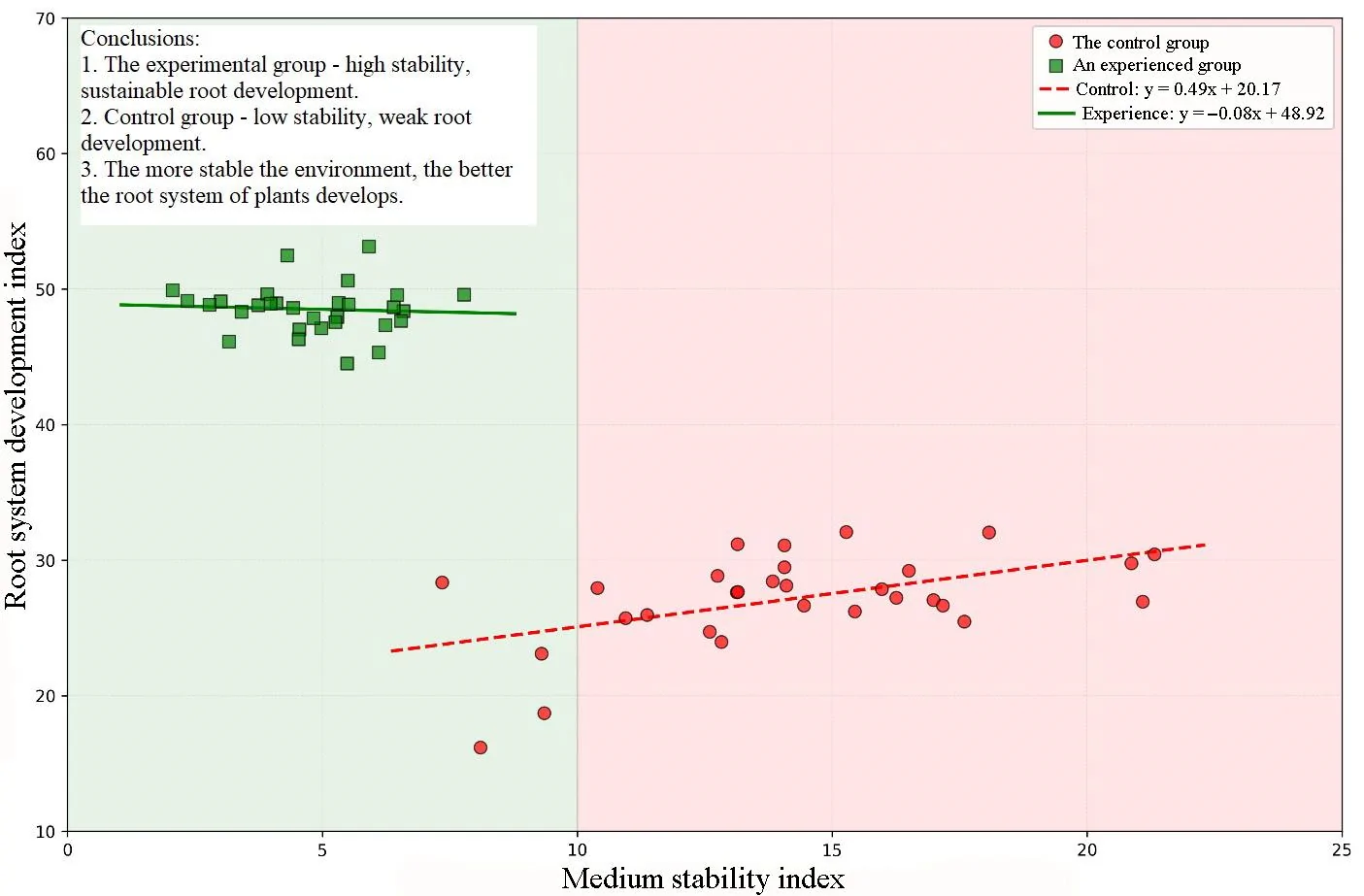
Correlation between environmental stability and root system development in strawberry rosettes.

Plant physiology. The total chlorophyll content in the leaves of plants grown in the composite medium was 18% higher. Plants in the experimental group had dark green leaves and shortened, thickened petioles, whereas the control group exhibited slight anthocyanin coloration of the petioles and less intensive growth (Figure 16).

The scatter plot with regression lines shows the relationship between the medium stability index and the root system development index.

A second type of polymeric medium based on pure potassium polyacrylate hydrogel (B 415 K) without vermicompost was tested. This medium was prepared using a similar procedure: 4.00 ± 0.05 g of dry medium-fraction hydrogel (0.5–1.5 mm) was hydrated in 100 mL of deionized water, and excess free water was removed after complete swelling. The resulting gel mass without organic additives was used as the second experimental variant (hydrogel).

**Figure 17:**
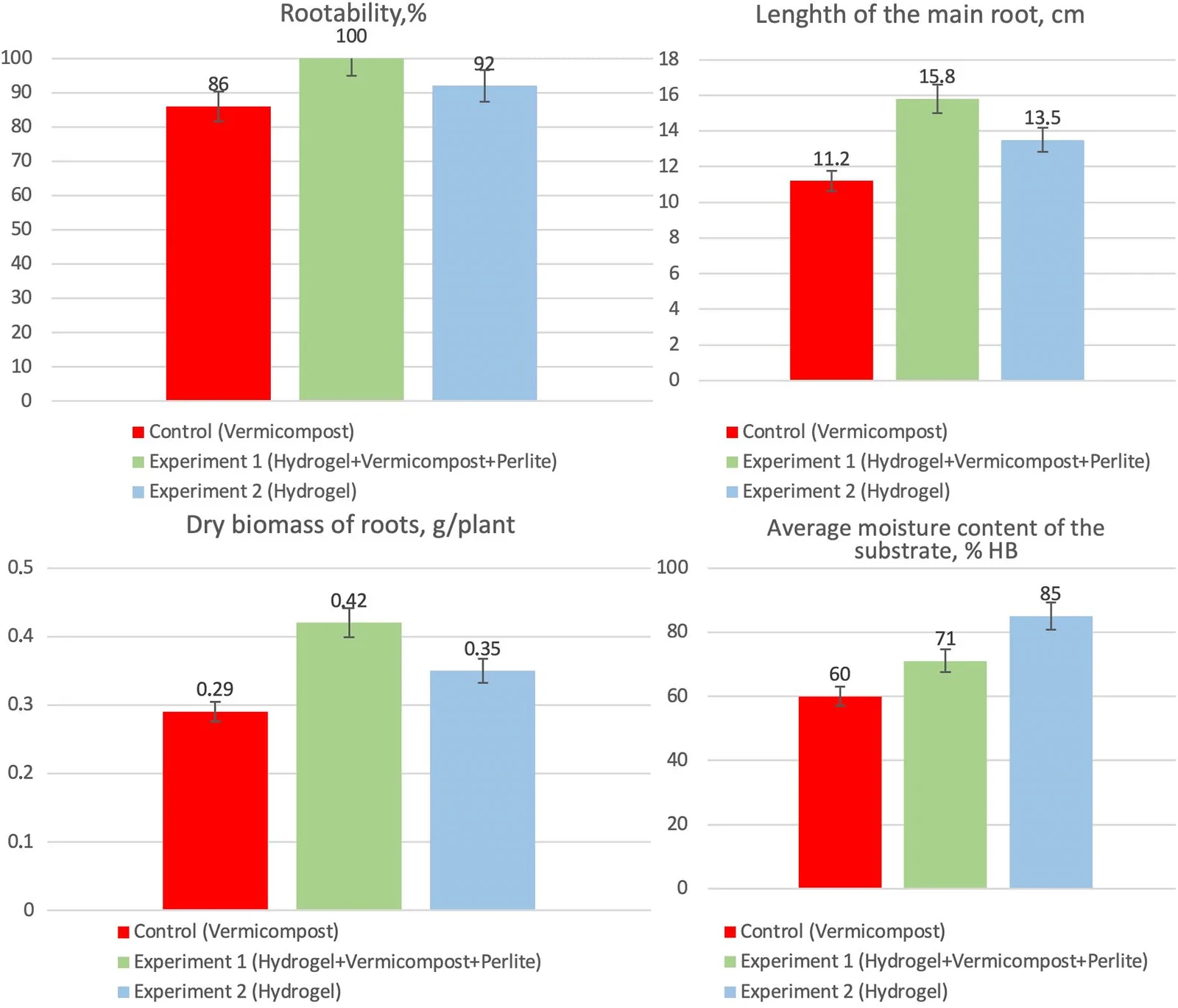
Comparative effectiveness of different types of substrates for strawberry rooting.

The experiment included three variants: (1) Control (optimized substrate: peat: perlite: vermicompost—50:30:20); (2) Experiment 1 (composite medium hydrogel + vermicompost + perlite); (3) Experiment 2 (medium consisting of pure hydrated polyacrylate gel without additives—hydrogel) (Figure 17).

The bar chart presents the key indicators of strawberry rosette rooting in the three substrate variants after 21 days of the experiment. The graph clearly demonstrates that the composite substrate (hydrogel + vermicompost + perlite) showed the best results across all parameters, whereas the pure hydrogel-based substrate, although superior to the control in terms of water retention, was inferior to the composite medium in root system development and plant biomass.

The observed differences between the three variants can be interpreted in terms of the functional properties of the polymeric media: the composite medium combines the water-retaining and ion-exchange functions of the hydrogel with the aeration provided by perlite and the organic matrix of vermicompost, whereas pure hydrogel provides only water retention and pure vermicompost lacks structural stability and aeration.

### 3.4. Field Validation of the Composite Polymeric Medium

The field experiment conducted in open ground using a manual soil auger for the application of the composite medium to the root zone confirmed the effectiveness of the developed material under real conditions. Table 2 presents the main morphophysiological parameters of ‘Alba’ strawberry plants on day 21 after planting.

Application of the composite medium to the root zone using the soil auger ensured stable soil moisture at 65–70% HB throughout the observation period, whereas in the control, moisture decreased to 50–55% as early as 10–12 days after irrigation. This made it possible to reduce irrigation frequency by approximately 30% without adversely affecting plant condition.

Plant establishment in the experimental variant reached 96.7%, which was 18.4% higher than in the control. Plants grown in the composite medium had a significantly more highly branched root system: total root length increased 1.57-fold, while the number of higher-order lateral roots (third- and fourth-order) nearly doubled. Root and aboveground dry biomass exceeded the control values by 46% and 40%, respectively. The higher chlorophyll content (by 18%) indicates more active photosynthesis and the absence of stress-related disturbances in the pigment apparatus.

It is important to note that the concentration of exchangeable potassium in the soil solution of the experimental plots remained at 120–130 mg/L throughout all three weeks, whereas in the control it decreased from an initial value of approximately 95 to 58 mg/L. This supports the interpretation that the hydrogel functions as an ion-exchange reservoir, providing prolonged plant nutrition even under field conditions without additional potassium fertilization.

The field data obtained are fully consistent with the results of the laboratory experiment and demonstrate the practical applicability of the developed composite medium for sustainable production of strawberry planting material in open ground, particularly in regions with limited water resources.

## 4. Discussion

The experiments confirmed that the composite polymeric medium based on potassium polyacrylate hydrogel, vermicompost, and perlite significantly increases the rooting efficiency and primary growth of strawberry rosettes compared with both traditional organic substrates (pure vermicompost) and pure hydrogel. This is consistent with the concept that sustainable strawberry production in protected and substrate-based systems depends not only on the optimization of mineral nutrition and microclimate, but also on the creation of a stable rooting environment with a regulated water regime, pH buffering capacity, and nutrient availability.

From a materials science perspective, the observed differences between the three variants can be interpreted in terms of structure–property–performance relationships. The medium hydrogel fraction (0.5–1.5 mm) demonstrated the most pronounced Raman spectrum of organic compounds, indicating effective immobilization of the organic growth stimulants on the particle surface. This conclusion is further supported by quantitative batch adsorption experiments: the medium fraction showed the highest equilibrium adsorption capacities for thiamine, 42.5 ± 2.1 mg/g, and succinic acid, 38.2 ± 1.9 mg/g, with adsorption kinetics and isotherms confirming chemisorption-dominated uptake. This is attributed to the optimal balance between surface area, hydration rate, and structural integrity. The fine fraction, although highly hydrated, showed less pronounced sorption of organic compounds, while the coarse fraction was the least hydrated and least effective at retaining organic compounds. These results demonstrate that the hydrogel fraction is a critical design parameter for composite polymeric media. The structure–property–performance relationships established in this work provide a basis for the rational design of composite polymeric media with tunable water-retention and ion-exchange properties.

A further point is the difference between swelling measured in distilled water and in the actual rhizosphere. The swelling of potassium polyacrylate is governed by the osmotic pressure of counterions, polymer–solvent interactions, and elastic retraction, and is reduced by dissolved ions, especially multivalent cations such as Ca^2+^ and Mg^2+^, which form ionic cross-links, as well as by dissolved organic matter. Therefore, the swelling ratios reported here (23.4, 19.1, and 12.7 g/g for the fine, medium, and coarse fractions) should be regarded as intrinsic characteristics of the polyacrylate network rather than as direct predictions of swelling in soil. However, the functional performance of the composite medium was assessed in the presence of a real ionic and organic environment: vermicompost supplied dissolved organic matter and humic substances, and water retention and K^+^ release were monitored in the substrate extract. The stable moisture (68–72% of full water-holding capacity) and K^+^ concentration (120–135 mg/L) over 21 days thus reflect behavior under ionic conditions close to those of the rhizosphere, rather than in pure water. A systematic study of swelling and release as a function of ionic strength, Ca^2+^/Mg^2+^ concentration, and dissolved organic matter content remains to be performed and is identified as a direction for future work.

These findings are consistent with the broader trend in recent materials science toward multifunctional hydrogel systems for sustainable environmental and agricultural applications. Recent reviews on chitosan-based hydrogels have emphasized the importance of rational design of biopolymer networks with tunable adsorption and release functions, as well as their integration with organic and inorganic fillers to create composite materials with combined functionalities such as adsorption, nutrient delivery, and environmental remediation [36,37]. Similar principles of structure–property–performance relationships are now being extended to the rational nanoscale design of porous functional materials for next-generation energy and environmental technologies, where pore architecture, surface chemistry, and interfacial interactions govern the material performance [38]. The fraction-dependent behavior of the potassium polyacrylate hydrogel observed in the present study follows the same design logic, in which particle size, network cross-linking, and the presence of organic and mineral fillers determine the coupled water-retention and ion-exchange functions of the composite polymeric medium.

The main factor responsible for the advantage of the composite medium was the high stability of the water regime. In the “hydrogel + vermicompost + perlite” variant, moisture content was maintained within a narrow range of approximately 68–72% of the full water-holding capacity, whereas in the control it decreased to 55–60% between irrigations, causing short-term water stress. Due to the presence of perlite and hydrogel granules, the composite medium maintained high redox potential (Eh) values ranging from +150 to +220 mV and dissolved oxygen (O_2_) concentrations of 5.2–6.5 mg/L, which is crucial for root respiration and ion uptake. In the control, by day 14, Eh decreased to +80 to +120 mV, while O_2_ decreased to 3.8–4.5 mg/L, indicating temporary hypoxia and confirmed by a decrease in the proportion of actively branching roots. The stability of electrical conductivity (EC) in the composite medium (1.15–1.25 mS/cm) indicates the absence of nutrient depletion, whereas in the control, EC decreased from 1.20 to 0.85 mS/cm by day 21.

Against the background of a stabilized water supply, rooting in the composite medium reached 100%, compared with 86% in the control and 92% in the pure hydrogel. At the same time, the composite medium resulted in increased main root length, total root system length, and the number of third- and fourth-order roots, while root dry biomass was 45% higher than in the control and 20–25% higher than in the pure hydrogel variant.

An important result was the confirmation that the composite medium not only provides a stable water regime but also ensures sustained potassium release while maintaining an optimal pH range. Over the 21-day period, the concentration of exchangeable K^+^ in the extract from the composite medium remained at 120–135 mg/L, whereas in pure vermicompost it decreased from 100 to 65 mg/L. This indicates a gradual depletion of available potassium in the control and, conversely, the presence of an ion-exchange reservoir in the hydrogel. A similar effect of prolonged macronutrient release in the presence of a polymer matrix has previously been described for agrohydrogels containing mineral salts, where ion exchange and diffusion from the gel contribute to stabilizing the dynamics of nutrient supply to plants and reducing leaching. In this case, the pH in the composite medium was maintained within the range of 6.1–6.3, which is optimal for strawberries, whereas pure vermicompost showed a shift toward a more acidic range, reaching 5.7, which potentially limits the availability of certain cations and increases the risk of root rot.

The relative contribution of vermicompost and of the polyacrylate network to the K^+^ budget requires clarification. Vermicompost contains exchangeable K^+^ and can release it during rooting, so its contribution cannot be neglected. However, three observations indicate that the hydrogel is the dominant factor. First, the same amount of vermicompost was present in both the composite medium and the control, yet only the composite medium maintained stable K^+^ at 120–135 mg/L, whereas the control decreased from 100 to 65 mg/L. Second, the pure hydrogel variant, which contained no vermicompost, maintained K^+^ at 90–110 mg/L, confirming that the polyacrylate network alone sustains K^+^ availability through ion exchange. Third, the K^+^ curve of the composite medium showed only minor fluctuations, consistent with a buffered ion-exchange mechanism rather than with progressive release from organic matter mineralization. A dedicated vermicompost + perlite control without hydrogel would allow the contribution of the organic filler to be quantified more precisely and is identified as a direction for future work.

The ion-exchange behavior of the potassium polyacrylate hydrogel is a key functional property of the developed polymeric medium. The polymer network contains carboxylate groups that can exchange K^+^ ions with the surrounding solution, acting as a buffer reservoir. This explains why the K^+^ concentration in the composite medium remained stable over 21 days, whereas in the control it decreased due to leaching and plant uptake. The sustained release of K^+^ from the polymer matrix, combined with the water retention function, makes the composite medium a multifunctional polymeric material for root-zone application.

The service life of the polyacrylate network is an important practical parameter for application frequency. Cross-linked potassium polyacrylate is not readily biodegradable and loses its functional properties in soil mainly through physical fragmentation, repeated swelling–deswelling cycles, and slow microbial and UV-assisted chain scission, with a reported service life of about 2–5 years under conventional tillage [35]. In the present study, water retention and K^+^ release were characterized only during the initial 21-day rooting stage, so the reported 21-day performance characterizes the rooting stage rather than the full service life of the material. Long-term field trials over several cultivation cycles are therefore required to quantify the loss of swelling capacity and the decrease in water retention and K^+^ release after repeated use. Based on the available literature and the results obtained, reapplication of the composite medium once every 2–5 growing seasons can be provisionally recommended, subject to refinement in future multi-year experiments.

The developed composite medium complies with SDG 6 (improving water-use efficiency), SDG 12 (reducing fertilizer leaching and using organic waste—vermicompost), and SDG 15 (preventing substrate acidification and preserving soil fertility). The stable water regime and prolonged potassium release make it possible to reduce irrigation and fertilizer application at the rooting stage, thereby reducing the environmental footprint of strawberry planting material production.

The economic feasibility of the composite medium depends on the balance between the cost of the hydrogel and the savings achieved during rooting. The hydrogel is the main cost component, whereas vermicompost and perlite are inexpensive and constitute the bulk of the medium. Because the hydrogel represents only a small mass fraction of the final mixture, the cost increment per plant relative to a conventional peat-based substrate is moderate. This increment is offset by reduced irrigation frequency, elimination of starter potassium fertilization, and higher survival rate, which together lower water, labor, and replanting costs. A full life-cycle cost analysis, including hydrogel reapplication every few growing seasons, remains to be performed, but the economic effect for strawberry nursery production is expected to be positive.

Field testing using a manual soil auger confirmed that the advantages of the composite medium identified under laboratory conditions are maintained under real agroecological conditions in open ground. Despite natural fluctuations in temperature and humidity, the application of the hydrogel composite to the root zone ensured stabilization of the soil moisture regime at 65–70% of the full water-holding capacity, whereas in the control, moisture decreased to 50–55% by the middle of the observation period. This made it possible to reduce irrigation frequency by 30% without loss of productivity. The survival rate of rosettes under field conditions reached 96.7%, which was only slightly lower than the laboratory value (100%) and significantly higher than the control value (78.3%). The field data on the increase in total root length (1.57-fold), the number of higher-order lateral roots (nearly twofold), and dry biomass (by 40–46%) are fully consistent with the results obtained in the climate chamber. Of particular significance is the confirmation of the prolonged potassium-release effect: the concentration of exchangeable K^+^ in the rhizosphere of the experimental plants remained at 120–130 mg/L throughout the three-week period, whereas in the control it decreased from approximately 95 to 58 mg/L. Thus, the developed composite medium demonstrated its effectiveness not only under controlled conditions but also under field conditions, opening up prospects for its implementation in nursery cultivation and organic farming, particularly in arid regions. It should be noted that the field experiment did not include a separate control variant with vermicompost and perlite only, which is a limitation of the present field validation and should be addressed in future field trials to isolate the incremental contribution of the hydrogel under open-ground conditions. Another limitation is that only the ‘Alba’ cultivar was tested. Strawberry cultivars differ considerably in root vigor, root system architecture, and nutrient demand, particularly in potassium and calcium requirements during flowering and fruit filling, which may affect the optimal hydrogel fraction, the hydrogel: vermicompost: perlite ratio, and the irrigation regime. The composite medium developed here is based on functional design principles rather than on cultivar-specific tuning: the water-retention and ion-exchange functions are provided by the polyacrylate network and are therefore not cultivar-specific, whereas the organic and mineral fillers provide aeration and microbial support that are also largely cultivar-independent. For this reason, the medium can be expected to be applicable to other major cultivated strawberry cultivars, but the component ratio and irrigation thresholds may require adjustment. Validation on a wider set of cultivars, including day-neutral and short-day types with different root vigor, is therefore recommended as a direction for future work.

## 5. Conclusions

This study evaluated the effects of a composite substrate based on potassium polyacrylate hydrogel (particle size: 0.5–1.5 mm), vermicompost, and perlite on the rooting and early growth of ‘Alba’ garden strawberry (*Fragaria* × *ananassa*) rosettes. In addition, the fraction-dependent properties of the hydrogel and the ion-exchange behavior of the composite polymeric media were characterized. The main conclusions are as follows:

1. The fraction-dependent sorption of organic growth stimulants was confirmed by Raman spectroscopy: the medium hydrogel fraction demonstrated the most pronounced bands at 1086, 710, 673, 531, 282, and 157 cm^−1^, assigned to C–O and C–C stretching vibrations of the immobilized heteroauxin and to carboxylate groups of the polyacrylate network. The coarse fraction showed no clearly resolved bands in the 600–1100 cm^−1^ region, indicating lower sorption capacity. These results demonstrate that the hydrogel fraction is a critical design parameter for composite polymeric media. Quantitative adsorption kinetics and isotherms confirmed that the medium fraction has the highest adsorption capacity for thiamine and succinic acid, providing direct quantitative support for the Raman-based conclusion.
2. The swelling behavior of the hydrogel fractions followed the order: fine (23.4 ± 0.8 g/g) > medium (19.1 ± 0.6 g/g) > coarse (12.7 ± 0.9 g/g). Although the fine fraction exhibited the highest swelling ratio, the medium fraction provided the optimal balance between hydration, structural integrity, and sorption of bioactive compounds, and was therefore selected for the design of the composite polymeric medium.
3. The composite polymeric medium functioned as a coupled water-retention and ion-exchange system: exchangeable K^+^ remained at 120–135 mg/L over 21 days without additional mineral fertilization, while the control decreased from 100 to 65 mg/L. The release profile is consistent with the ion-exchange mechanism controlled by carboxylate groups of the polyacrylate network.
4. The developed composite medium (hydrogel:vermicompost:perlite = 1:40:20 by dry weight) ensures a stable water regime: substrate moisture is maintained within a narrow range of 68–72% of the full water-holding capacity throughout the experiment (21 days), whereas in the control (optimized commercial substrate, peat:perlite:vermicompost = 50:30:20), moisture decreases to 55–60% of the full water-holding capacity, resulting in short-term water stress in the plants.
5. The composite medium promotes 100% rooting of strawberry rosettes (86% in the control and 92% in pure hydrogel). At the same time, total root system length increases 1.6-fold, the number of third- and fourth-order lateral roots increases 1.8–2.1-fold, and root dry biomass increases by 45% compared with the control.
6. The composite medium provides optimal pH buffering (6.1–6.3) and also maintains high values of redox potential (Eh) from +150 to +220 mV and dissolved oxygen (O_2_) 5.2–6.5 mg/L, which may reduce the risk of root hypoxia and promote root branching. The electrical conductivity (EC) in the composite remains stable (1.15–1.25 ms/cm), while in the control it decreases from 1.20 to 0.85 ms/cm by day 21, and the pH shifts to the acidic side to 5.7.
7. Plants grown in the composite substrate are characterized by a higher physiological status: the content of chlorophylls *a* and *b* in the leaves is 18% higher than in the control substrate, and the leaf blades exhibit an intense dark-green coloration without signs of anthocyanin-related stress.
8. From an ecological and agronomic perspective, the proposed composite polymeric medium complies with the principles of sustainable agriculture: it reduces the need for frequent irrigation (water savings of approximately 30%), decreases nutrient leaching, completely eliminates the need for initial potassium fertilizer application at the rooting stage, and may be recommended for use in protected cultivation systems and nurseries, particularly in regions with limited water resources. The estimated service life of the hydrogel is 2–5 growing seasons.
9. Practical recommendation: for the preparation of the composite polymeric medium, it is advisable to use a medium-fraction hydrogel (0.5–1.5 mm), which, according to Raman spectroscopy data, provides an optimal balance between hydration, structural integrity, and the ability to immobilize organic growth stimulants. For aeration, 20% perlite should be added.
10. Field trials using a manual auger have demonstrated the practical applicability of the composite medium under open-ground conditions. The survival rate of rosettes reached 96.7%, and all morphophysiological parameters (root length, biomass, chlorophyll content) significantly exceeded the control. Moisture stability and prolonged potassium nutrition made it possible to reduce irrigation frequency by 30% without the application of starter mineral fertilizers, which confirms the environmental and economic feasibility of using the developed material under production conditions.

## Figures and Tables

**Table 1:** The results of the elemental analysis of polymer hydrogel by atomic emission spectroscopy.

Sample No.	Element	C, ppm	Standard Deviation, %
Large	Na	24.2	1.54
	K	167.4	1.75
	Ca	152.7	7.01
	Mg	38.2	3.88
Average	Na	22.1	3.72
	K	164.6	0.86
	Ca	155.3	0.92
	Mg	35.9	5.07
Small	Na	19.8	4.96
	K	147.7	0.79
	Ca	143.7	0.81
	Mg	30.5	4.92
Hoagland Rr.	On	30.3	1.38
	K	244.0	0.64
	Ca	217.2	6.55
	Mg	54.9	1.96

**Table 2:** Comparative assessment of growth parameters and physiological status of plants in the field experiment (M ± SE, *n* = 60).

Parameter	Control (Unamended Soil)	Composite Medium (Hydrogel + Vermicompost + Perlite)
Survival rate, %	78.3 ± 2.1	96.7 ± 1.5 *
Main root length, cm	9.4 ± 0.8	14.2 ± 0.9 *
Total root system length, cm	48.6 ± 3.2	76.4 ± 4.1 *
Number of lateral roots of 3rd–4th orders, units	12.5 ± 1.1	23.8 ± 1.6 *
Root dry biomass, g/plant	0.28 ± 0.02	0.41 ± 0.03 *
Shoot dry biomass, g/plant	0.52 ± 0.04	0.73 ± 0.05 *
Total chlorophyll *a* + *b* content, mg/g fresh weight	1.82 ± 0.06	2.15 ± 0.07 *
Exchangeable K^+^ concentration in soil solution, mg/L (day 21)	58.4 ± 4.2	127.6 ± 5.8 *

*—differences are significant at *p* < 0.05 (ANOVA).

## Data Availability

The original contributions presented in the study are included in the article; further inquiries can be directed to the corresponding author.
